# Discovery of Putative Dual Inhibitor of Tubulin and EGFR by Phenotypic Approach on LASSBio-1586 Homologs

**DOI:** 10.3390/ph15080913

**Published:** 2022-07-23

**Authors:** Gisele Barbosa, Luis Gabriel Valdivieso Gelves, Caroline Marques Xavier Costa, Lucas Silva Franco, João Alberto Lins de Lima, Cristiane Aparecida-Silva, John Douglas Teixeira, Claudia dos Santos Mermelstein, Eliezer J. Barreiro, Lidia Moreira Lima

**Affiliations:** 1 Laboratório de Avaliação e Síntese de Substâncias Bioativas (LASSBio^®^), Instituto Nacional de Ciência e Tecnologia de Fármacos e Medicamentos (INCT-INOFAR), Universidade Federal do Rio de Janeiro, Rio de Janeiro 21941-902, RJ, Brazil; bgiselejf@gmail.com (G.B.); luisga011@hotmail.com (L.G.V.G.); carolinemxc@gmail.com (C.M.X.C.); silvafrancolucas@gmail.com (L.S.F.); albertinho_49@hotmail.com (J.A.L.d.L.); cristianesilva486@gmail.com (C.A.-S.); ejbarreiro@ccsdecania.ufrj.br (E.J.B.); 2 Programa de Pós-graduação em Farmacologia e Química Medicinal, Instituto de Ciências Biomédicas, Universidade Federal do Rio de Janeiro, Rio de Janeiro 21941-902, RJ, Brazil; 3 Laboratório de Diferenciação Muscular e Citoesqueleto, Instituto de Ciências Biomédicas, Universidade Federal do Rio de Janeiro, Rio de Janeiro 21941-902, RJ, Brazil; teixeira.john@gmail.com (J.D.T.); mermelstein@histo.ufrj.br (C.d.S.M.)

**Keywords:** tubulin, microtubules, homologs, EGFR, phenotypic assay

## Abstract

Combretastatin A-4 (CA-4, **1**) is an antimicrotubule agent used as a prototype for the design of several synthetic analogues with anti-tubulin activity, such as LASSBio-1586 (**2**). A series of branched and unbranched homologs of the lead-compound **2**, and vinyl, ethinyl and benzyl analogues, were designed and synthesized. A comparison between the cytotoxic effect of these homologs and **2** on different human tumor cell lines was performed from a cell viability study using MTT with 48 h and 72 h incubations. In general, the compounds were less potent than CA-4, showing CC_50_ values ranging from 0.030 μM to 7.53 μM (MTT at 72 h) and 0.096 μM to 8.768 μM (MTT at 48 h). The antimitotic effect of the target compounds was demonstrated by cell cycle analysis through flow cytometry, and the cellular mechanism of cytotoxicity was determined by immunofluorescence. While the benzyl homolog **10** (LASSBio-2070) was shown to be a microtubule stabilizer, the lead-compound **2** (LASSBio-1586) and the methylated homolog **3** (LASSBio-1735) had microtubule destabilizing behavior. Molecular docking studies were performed on tubulin protein to investigate their binding mode on colchicine and taxane domain. Surprisingly, the benzyl homolog **10** was able to modulate EGFR phosphorylate activity in a phenotypic model. These data suggest LASSBio-2070 (**10**) as a putative dual inhibitor of tubulin and EGFR. Its binding mode with EGFR was determined by molecular docking and may be useful in lead-optimization initiatives.

## 1. Introduction

Antimicrotubule agents are often used in antimicrobial therapy because their modularity and microtubule dynamics agents interfere with spindle capacity, pausing the cell cycle at the G2/M checkpoint. Therefore, the cell cycle comes to arrest in rapidly proliferating cancer cells leading to apoptosis by stabilizing or destabilizing the microtubule polymer [1,2]. Antimitotic drugs affect microtubule dynamics upon binding to one of the three established drug domains (taxane, vinca alkaloid, or colchicine site) on tubulin, the building blocks of microtubule. Compounds capable of binding to the colchicine site inhibit microtubule formation and prevent a conformational change in tubulin necessary for polymerization [2,3,4]. Combretastatin A-4 (CA-4) is a classic example of a natural product able to bind to the colchicine site. Due to its solubility limitation, the disodium phosphate monoester of CA-4 (i.e., CA-4P) was developed to treat anaplastic thyroid cancer in combination with other anticancer drugs [5].

Several synthetic analogues of CA-4 (**1**) have been developed, aiming to maintain the broad-spectrum cytotoxicity profile of the natural compound and to circumvent its low water solubility and chemical instability, due to the inter-conversion of the olefinic double bond from the more active cis to less active trans-conformation [6]. LASSBio-1586 (**2**) has been described as a stable CA-4 analogue. This acylhydrazone derivative displayed excellent results in tumor cell lines and in vivo cytotoxic activity, being able to decrease proliferation of the SF-295 and HCT-116 cell lines in the Hollow Fiber Assay. The ability of LASSBio-1586 to inhibit β-tubulin was confirmed, revealing this compound as a new anti-tubulin agent structurally correlated to CA-4. In attempt to establish the structural activity relationship of LASSBio-1586, Amaral and coworkers demonstrated the crucial role of the acylhydrazone framework and the trimethoxyphenyl subunit for cytotoxic activity [7,8,9]. Among the structural modifications introduced in the acylhydrazone subunit, the *N*-methylation of the *sp3* nitrogen proved to be tolerated, resulting in compound LASSBio-1735 (**3**) with cytotoxic potency superior to LASSBio-1586 (**2**) against HL-60 cell lines [7].

Having in mind the role of homologation as an important strategy for molecular modification [10], and assuming the necessity to optimize LASSBio-1586 (**2**) cytotoxic activity, this paper describes the design and synthesis of new homologs (**4**–**12**) of compounds **2** and **3**, their comparative cytotoxic profile, cellular mechanism of action and their binding mode with target proteins by molecular docking.

**Figure 1:**
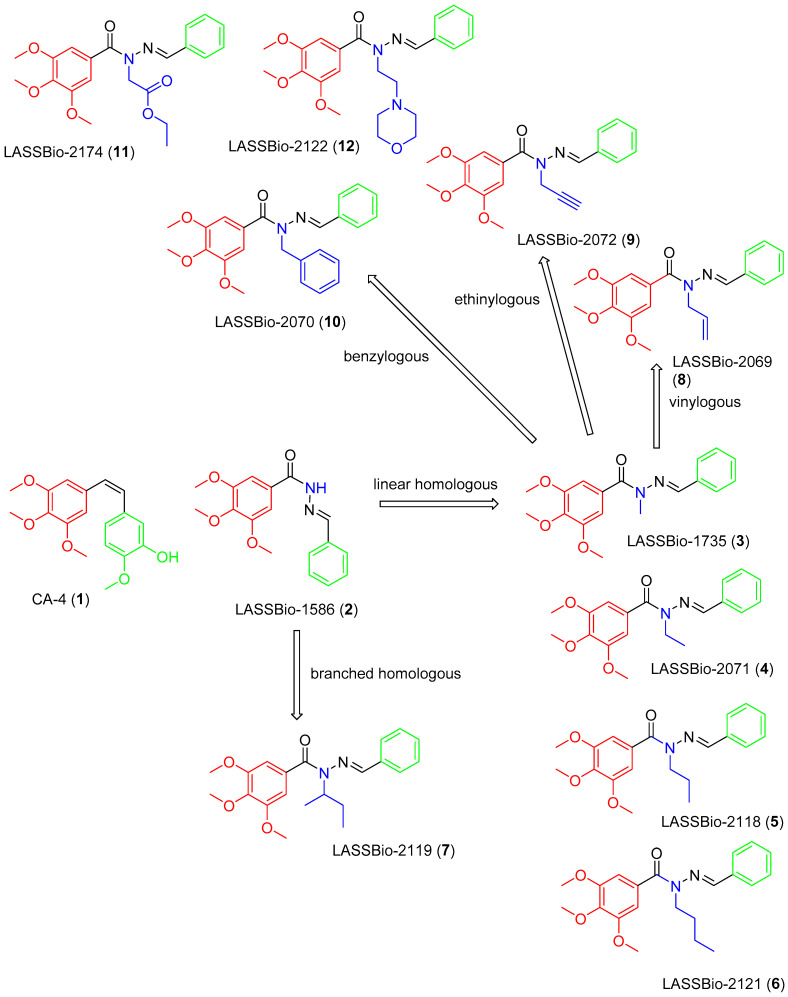
Structures of CA-4 (**1**), LASSBio-1586 (**2**) and compounds **3**–**12** designed using homologation strategy on the nitrogen of *N*-acylhydrazone framework.

The design concept of the linear (**3**–**6**) and branched (**7**) homologs, as well as the vinyl (**8**), ethinyl (**9**) and phenyl (**10**) homologs, are shown in Figure 1. To explore an improvement in aqueous solubility, compounds bearing the carbethoxy (**11**) and ethyl-morpholine (**12**) subunits were also designed and are displayed in Figure 1.

## 2. Results and Discussion

### 2.1. Chemistry

**Figure 2:**
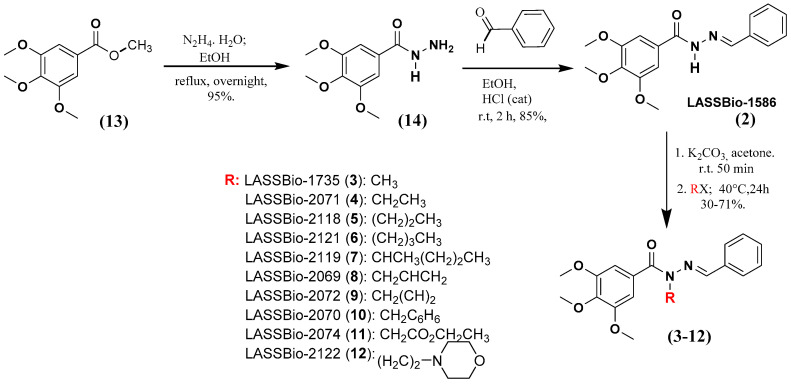
Synthetic methodology to obtain LASSBio-1586 (**2**) homologs and analogues from the starting material **13**.

The homologs of LASSBio-1586 (**2**) and LASSBio-1735 (**3**) were synthetized using the previous methodology described by Amaral and coworkers [7] and summarized in Figure 2. The key synthetic step involved the selective *N*-alkylation of the *sp3* nitrogen of the intermediate **2** (LASSBio-1586). All the compounds were characterized by ^1^H NMR, ^13^C NMR, ^13^C DEPT-135 NMR, IR and mass spectroscopy, and their purity was determined by HPLC with a reverse-phase column (C-18) with an isocratic mobile phase in the same system of CH_3_CN: H_2_O (60:40 *v*/*v*).

The presence of the characteristic signal of the imine carbon (N=**C**H) in the ^13^C NMR spectra of compounds **3**–**12**, in addition to the presence of the characteristic band of the carbonyl group (Ar**CO**NRN=CHR) in the infrared spectra, allowed confirmation of selective *N*-alkylation, ruling out the hypothesis of *C*- or *O*-alkylated products (Figure 2), as it had already been detailed by Amaral and coworkers [7] to LASSBio-1735 (**3**). The physicochemical properties of the compounds reported in these studies are summarized in Table 1.

### 2.2. Pharmacological Experiments

#### 2.2.1. Cellular Cytotoxicity

The cytotoxic activity of compounds **3**–**13** was determined based on an MTT assay [11] against different human tumor cell lines, using LASSBio-1586 (**2**), LASSBio-1735 (**3**), CA-4 (**1**) and pelitinib as standards (Table 2). To determine the cytotoxic selectivity index, compounds were also evaluated toward human lymphocytes (Table 3). 

As shown in Table 2, apart from LASSBio-2119 (**8**), all compounds exhibited moderate to high cytotoxic potency with CC_50_ (concentration that reduced the proliferation of cells by 50%) values of 39 nM to 8.5 μM. As expected, CA-4 (**1**) exhibited cytotoxic potency in the nanomolar range for all cell lines studied (except for MCF-7). While pelitinib, a non-selective irreversible EGFR (Epidermal Growth Factor Receptor) inhibitor, showed potency ranging in the nanomolar to low micromolar range, with emphasis on its effect on lung tumor lines overexpressing EGFR (such as LoVo, H292 and H1975).

The impact of homologation strategy to cytotoxic activity of LASSBio-1586 (**2**) analogues can be seen by the comparison between the potency of compounds **3**–**12**. As demonstrated in Table 1, although the linear homologs (**4**–**6**) were active, none of them were better than the *N*-methylated compound (**3**), suggesting that increasing the carbon chain does not result in optimizing the cytotoxic effect. The homologation strategy by adding fragments with *sp^2^* or *sp* hybridization resulted in compounds **8**–**10**. In general, the vinyl (**8**), ethinyl (**9**) and phenyl (**10**) homologs displayed similar cytotoxic potency, but still being less active than LASSBio-1735 (**3**). 

In fact, the *N*-methylated homolog **3** (LASSBio-1735) had a high cytotoxic potency against acute myeloid leukemia (HL-60 cell lines), being 53 times more potent (CC_50_ 39 nM) than LASSBio-1586 (CC_50_ 2.08 µM), corroborating our previous report [7]. We demonstrated that LASSBio-1735 (**3**) also showed great cytotoxic potency in breast adenocarcinoma (MCF-7) with CC_50_ of 0.28 µM. These data reveal that this homologous was 27 and 31 times more potent on MCF-7 than LASSBio-1586 (CC_50_ 7.53 µM) and CA-4 (**1**) (CC_50_ 8.58 µM), respectively (Table 3). 

MCF-7 is a cell line known as a multidrug-resistant breast cancer cell line. Its resistant phenotype is attributed to the overexpression of the multiple drug carrier P glycoprotein (Pgp) [12,13]. Pgp is an efflux transmembrane protein, dependent on ATP, with broad substrate specificity, and known as a multi-drug resistance protein (MDR). Substrates of Pgp have compromised cytotoxic efficacy on MDR phenotype tumors. Tumor resistance to antitubulin drugs is believed to result from Pgp drug-efflux [14]. Therefore, the observation that all compounds were more potent than CA-4 (**1**) on the MCF-7 cell line, emerged as quite promising result (Table 2), although the ability of the compounds to act as substrates for Pgp still needs to be investigated comparatively to CA-4 (**1**).

To establish the cytotoxic selective index (SI) of LASSBio-1586 homologs, they were studied on human lymphocyte cell lines (GM16000), using the MTT assay. The SI was calculated by the ratio between the CC_50_ on tumor and non-tumor cell lines [15]. Although there is no consensus, values of SI ≥ 10 is considered satisfactory to assign safety to a new cytotoxic agent. This value means that the compound is more than ten times more cytotoxic to the tumor cell line than to normal cell line. 

As depicted in Table 3, like CA-4 (**1**), most of the tested compounds showed low SI (SI< 10). For the three tumor lines studied, pelitinib exhibited the best SI (Table 3), probably due to the fact that it is a target-directed cytotoxic agent with a targeted effect on cells overexpressing EGFR. The worst cytotoxic selectivity profile found for CA-4 (**1**), LASSBio-1586 (**2**) and their homologs (**3**–**12**) may be associated with their nonspecific cytotoxic mechanism of action, acting as antimicrotubule or anti-tubulin agents.

The calculated SI for compounds **2**–**12** revealed that two homologs (**4** and **11**) stood out by showing SI higher than 10. LASSBio-2071 (**4**) exhibited SI of 22.2, 17.0 and 13.3 to HL60, LoVo and PC9 cell lines, respectively, while LASSBio-2074 (**11**) displayed an SI of 33.3, 39.5 and 20.8. against HL60, LoVo and PC9 tumor cell lines (Table 3). The lead compound **2** showed a favorable cytotoxic selectivity index only for the LoVo cell line (SI = 39).

The cytotoxic potencies of compounds **3**–**12** were also studied in an MTT assay of 48 h, in a comparative manner to LASSBio-1586 (**2**), CA-4 (**1**) and pelitinib standards. All compounds tested were less potent in the 48 h MTT (Table 4) when compared to the 72 h MTT (Table 2). Compounds **3** (LASSBio-1735) and **4** (LASSBio-2071) showed more promise than the others. The former showed CC_50_ values of 0.096 µM, 0.152 µM and 0.479 against the H1975, H292 and LoVo cell lines, respectively. The latter presented a CC_50_ of 0.903 µM, 0.147 µM and 0.244 µM on H1975, H292 and PC9.

SI values in an MTT assay of 48h were also determined (Supplementary Material) and only LASSBio-2070 (**10**) showed favorable SI, although only for two tumor cell lines (H292 and PC9). All others, including the standard drugs, exhibited SI values less than 10.

The excellent cytotoxic effect on MCF-7 observed for the MTT assay at 72 h of incubation was not observed when the experiment was performed at 48 h. Considering the great cytotoxic potency of LASSBio-1735 (**3**) on lung adenocarcinoma cell lines, including H1975 (the T790M-positive cell line that harbors the EGFR^L858R/T790M^ double mutation), both at 72 h and 48 h MTT, this compound was selected for further study. In parallel, we also chose LASSBio-2070 (**10**) that showed good cytotoxic potency at 48 h MTT, allowing us to compare the impact of homologation by a phenyl introduction on the cytotoxic cellular mechanism of LASSBio-1735 (**3**).

#### 2.2.2. Flow Cytometry Analysis

**Figure 3:**
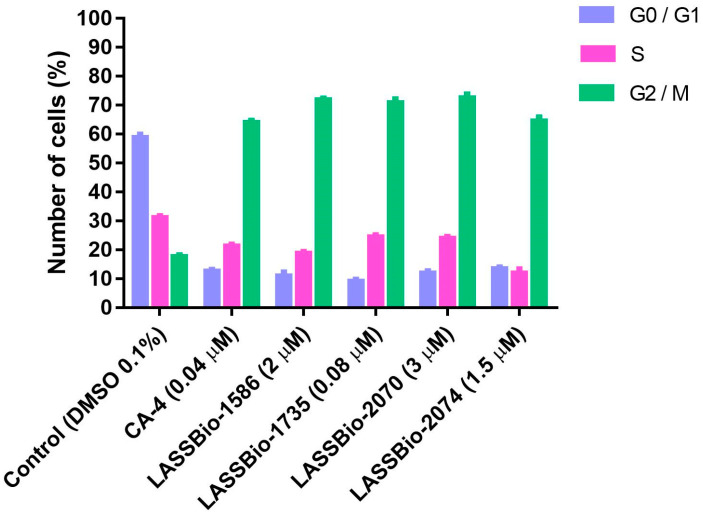
Effects of compounds treatment on G2/M phase arrest in H1975 cells. The cell cycle distribution after 48 h of treatment with CA-4 (**1**, 0.04 µM), LASSBio-1586 (**2**, 2.0 µM), LASSBio-1735 (**3**, 0.08 µM), LASSBio-2070 (**10**, 3.0 µM) and LASSBio-2074 (**11**, 1.5 µM), compared with the control (DMSO 0.1%), was measured using flow cytometry. The data are presented as mean values ± SD, obtained by at least three independent experiments performed in triplicate, the data being compared with the negative control by ANOVA followed by the Newman Keuls test in all phases with *p* < 0.05.

Changes in the cell cycle are important aspect in tumorigenesis. Therefore, the identification of the steps related to inhibition of cell multiplication is an important process during the discovery of new antitumoral drug candidates [16,17,18]. Considering that CA-4 (**1**) and LASSBio-1586 (**2**) are antimitotic agents, we decided to investigate the ability of the new homologs **3**–**12** to interrupt M phase of the cell cycle, promoting mitotic catastrophe and leading the cell to apoptosis. Flow cytometry was used for cell cycle analysis to estimate the percentages of a cell population in the different phases of the cell cycle. The analysis was performed using the lung cancer cell line H1975 treated with CA-4 (**1**, 0.04 µM), LASSBio-1586 (**2**, 2.0 µM), LASSBio-1735 (**3**, 0.08 µM), LASSBio-2070 (**10**, 3.0 µM) and LASSBio-2074 (**11**, 1.5 µM). As depicted in Figure 3, the cell cycle distribution after treatment with the target compounds (at concentrations 10 times lower than their CC_50_ determined by measuring the cell viability using MTT assay after 72 h of incubation) revealed very similar behavior between the target compounds and the standard drugs. They decreased cells in phase G0/G1 and S and increased cells in phase G2/M (Figure 3). These results confirm their antimitotic profile and attest that the homologation strategy (by designing the methylated and benzylated homologs of LASSBio-1586) did not compromise the proposed cellular mechanism of action, although it did affect the cytotoxic potency.

**Figure 4:**
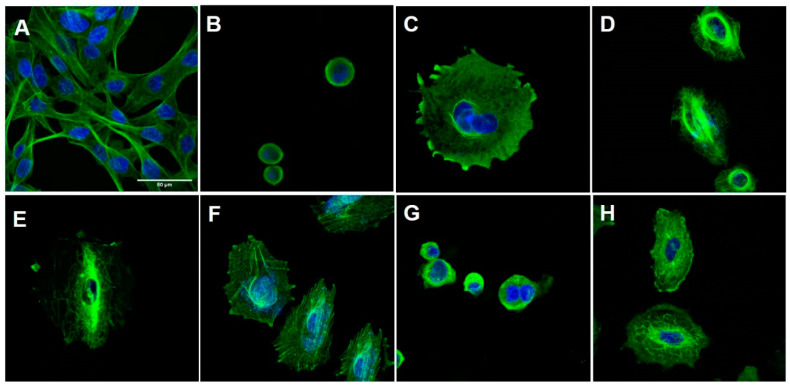
Immunofluorescence of H1975 cells. The cells were stained with an antibody against β-tubulin (green) and with DAPI (blue), viewed with a 63-fold objective, maintained with or without treatment for 48 h. (**A**) Negative control cells (DMSO 0,1%); (**B**) cells treated with CA-4 (**1**, 0.3 µM); (**C**) cells treated with vincristine (0.5 µM); (**D**) cells treated with taxol (0.1 µM); (**E**) cells treated with LASSBio-1586 (**2**, 10 µM); (**F**) cells treated with LASSBio-1735 (**3**, 0.5 µM); (**G**) cells treated with LASSBio-2070 (**10**, 4.5 µM); (**H**) cells treated with LASSBio-2074 (**11**, 20 µM). The data were obtained by at least two independent experiments performed in triplicate.

Antitubulin agents have been broadly classified into destabilizing or stabilizing agents, based on their interference with the dynamic balance of polymerization or depolymerization of microtubules [19]. To elucidate the cellular mechanism of action of LASSBio-1586 (**2**, 10 µM), LASSBio-1735 (**3**, 0.5 µM), LASSBio-2070 (**10**, 4.5 µM), and LASSBio-2074 (**11**, 20 µM), first identified as antimitotic agents (Figure 4), we performed immunofluorescence studies aiming to identify the morphological changes on the cytoskeleton of H1975 cells in the presence and absence of the target compounds. CA-4 (**1**, 0.3 µM), vincristine (0.5 µM) and taxol (0.1 µM) were used as standard controls. Compounds were assayed at a concentration two times higher than their CC_50_ to demonstrate their effect on the cells.

As shown in Figure 4, the H1975 cells in the control group had an elongated morphology, typical of this cell line. After treatment with CA-4 (**1**), vincristine and LASSBio-2070 (**10**) cell morphology was changed, assuming a rounded shape. The treatment with taxol, LASSBio-1586 (**2**), LASSBio-1735 (**3**) and LASSBio-2074 (**11**) resulted in cells with a more spread-out morphology. It was possible to observe a decrease in the number of cells for all fields treated with the compounds and the positive standards, probably indicating cell death from the high concentration of the drug used. The immunofluorescence studies demonstrated that CA-4 (**1**), vincristine and LASSBio-2070 (**10**) exhibited a typical response of microtubule destabilizing agents, or microtubules polymerization inhibitors, by binding to tubulin protein [20,21]. As shown in Figure 4, an increase in the density of microtubules (responsible for the shrinkage and rounding of cells) causing concentration of microtubules in a smaller cell area were observed after treatment of H1975 cells with CA-4 (**1**), vincristine and LASSBio-2070 (**10**). 

On the other hand, cells treated with LASSBio-1586 (**2**), LASSBio-1735 (**3**) and LASSBio-2074 (**11**) displayed a cell morphology similar to microtubule-stabilizing drugs such as taxol, with consequent depolymerization inhibition (Figure 4). In these cells, no drastic change in cell morphology was observed. However, microtubules were disorganized and rearranged irregularly in the cells, losing their dynamics, and unable to grow or decrease in size. These alterations, normally, lead to a loss of cellular function and, consequently, cell death [22,23]. Interestingly, these data contradict previous results published for LASSBio-1586 (**2**) that described this prototype as a structural analogue of CA-4 (**1**) capable of binding to the colchicine site of tubulin in silico studies [7].

Taken together, the results suggest that homologation has a significant impact on the binding domain of the compound to tubulin. Microtubule dynamics is controlled mainly by three established drug domains on tubulin, known as taxane, vinca alkaloid and colchicine sites. CA-4 (**1**) binds to tubulin at the domain known as the colchicine binding site. Binding to this site induces microtubule depolymerization in a mechanism typical of microtubule destabilizer agents. This mechanism were observed only for the benzyl homolog **10**, while LASSBio-1586 (**2**), the *N*-methyl homolog **3** and even the polar derivative **11** exhibited a cellular mechanism like microtubule stabilizer agents such as taxol, inducing polymerization of tubulin, stabilizing the polymer and preventing depolymerization [24,25,26,27]. To understand the molecular reasons why compounds **2**, **3** and **11** interact with the taxane domain, while compound **10** binds to the colchicine domain on tubulin, docking studies were performed and are discussed later.

**Figure 5:**
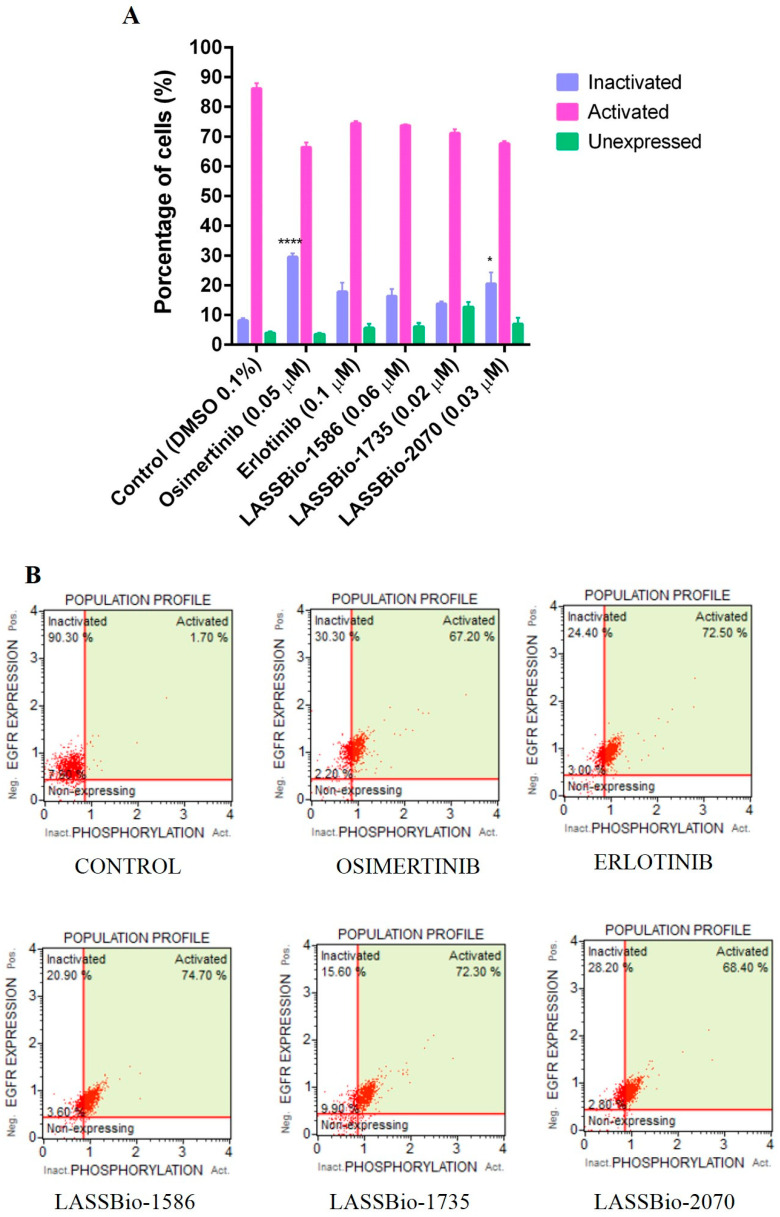
Determination of EGFR inhibition capacity in a PC-9 cell line (EGFR^L858R^) at 24 h. (**A**) Graphic representation of EGFR inhibition assay in a PC-9 cell line and with the respective percentages of cells containing activated, inactivated, and unexpressed EGFR. (**B**) Histograms from flow cytometry with demonstrative values. Evaluation of the cell cycle on PC-9 (EGFR^L858R^) at 24 h analyzed with a MUSE™ cell analyzer and compared to the control group. The data are presented as mean values ± SD, obtained by at least three independent experiments performed in triplicate, the data compared with the negative control by ANOVA followed by a multiple comparisons test in all phases with * *p* <0.05 and **** *p* < 0.0001.

Due to the good potency of the compounds on human lung adenocarcinoma cell lines overexpressing EGFR receptors, we decided to investigate the possibility of these compounds to modulate the activity of those receptors. To address this question, the selected compounds were assayed using the Guava Muse^®^ EGFR-RTK Activation Dual Detection Kit protocol. The experimental was performed on non-small cell lung cancer PC-9 cells that express the EGFR^L858R^ phenotype by flow cytometry analysis. Compounds were assayed using a single concentration based on their CC_50_ values on PC9 determined by the 48 h MTT method (Table 4). Osimertinib (EGFR irreversible inhibitor) and erlotinib (EGFR reversible inhibitor) were used as standard drugs, with concentrations of 0.05 µM and 0.1 µM, respectively. As demonstrated in Figure 5, erlotinib (a first-generation inhibitor of EGFR) as well as LASSBio-1586 (**2**, 0.06 µM), LASSBio-1735 (**3**, 0.02 µM) and LASSBio-2070 (**10**, 0.03 µM), exhibited a comparable EGFR inactivation index, although only the result for the benzyl homolog **10** was statistically significant. Osimertinib, a third-generation EGFR inhibitor that has been reported as a potent and selective inhibitor of EGFR mutations forms (i.e., L858R and L858R/T790M) [28], exhibited a superior EGFR inactivation index (30%). Taken together, the results indicate LASSBio-2070 (**10**) as able to modulate EGFR activity in a phenotypic model by inhibiting the phosphorylation of EGFR on PC-9 cell line (Figure 5). Therefore, its binding mode with EGFR was investigated by molecular docking and is further discussed later.

### 2.3. Molecular Docking Studies

As previously discussed, the experimental data obtained from the immunofluorescence studies (Figure 4) anticipated a distinct microtubule modulation profile between the prototype **2** and its methyl homolog **3** versus that found for the benzyl analogue **10**. To understand the molecular reasons for LASSBio-1586 (**2**) and LASSBio-1735 (**3**) profiles similar to taxol, and the behavior of LASSBio-2070 (**10**) similar to CA4, we performed a molecular docking study using tubulin structure co-crystallized with colchicine and taxol retrieved from the Protein Data Bank server (PDB ID: 5XIW and 6WVR). 

#### 2.3.1. Docking in the Colchicine Binding Site

**Figure 6:**
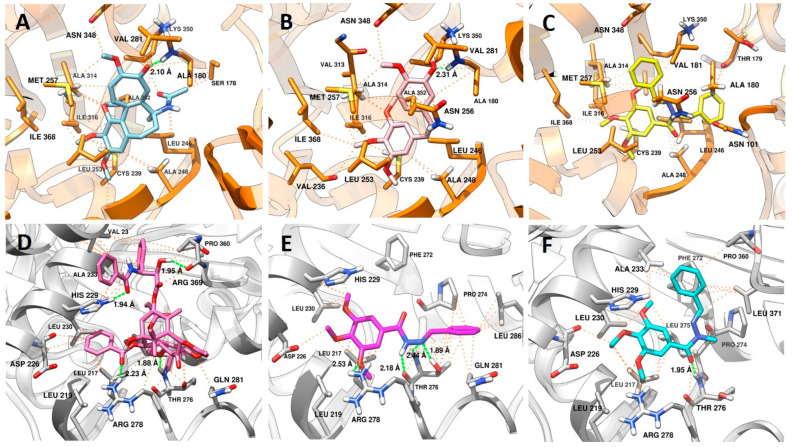
Analysis of the interaction mode in (**A**) colchicine in sky blue; (**B**) CA4 in pink; and (**C**) LASSBio-2070 (**11**) in yellow at the colchicine binding site (PDB: 5XIW). Protein carbon atoms are represented in orange, oxygen in red, nitrogen in blue and the distances between hydrogen bonds are represented by the green line. Hydrophobic interactions are represented by orange lines. Analysis of the interaction mode in (**D**) taxol in hot pink; (**E**) LASSBio-1586 (**3**) in magenta; (**F**) and LASSBio-1735 (**3**) in cyan at the taxol binding site (PDB: 6WVR). Protein carbon atoms are represented in gray, oxygen in red, nitrogen in blue and the distances between hydrogen bonds are represented by the green line. Hydrophobic interactions are represented by orange lines.

The colchicine binding site is mostly composed of hydrophobic amino acids (Ala180. Leu240, Ala248, Leu246, Leu250, Leu253, Val281, Ala314, Ile316, Val318, Ala352, Ile368), and highly polarizable amino acids, such as Cys239 and Met257. A steric clash, promoted by colchicine (Figure 6A) and analogues such a CA-4 (**1**) (Figure 6B), with amino acids residues of the α subunit, are important because this kind of interaction inhibits microtubule assembly in the intermediate domain of α and β subunits of tubulin, which is the region in which destabilizing agents interact [29,30].

Considering the binding mode of colchicine and CA-4 (**1**), where interactions with Cys239, Leu246, Leu253, Asn256 Met257, Ala314, Ile316, Lys350, Ala352, Ile368 amino acids are described as pharmacophoric [31,32,33], it is expected that ligands with non-polar groups would have stronger affinity for this binding site. In this way, the interactions promoted by LASSBio-2070 (**10**) (Figure 6C) at the colchicine binding site were efficient regarding the number of main interactions (Cys239, Leu246, Leu253, Asn256 Met257, Ala314, Ile316, Lys350, Ala352, Ile368), and of additional interactions (Asn101, Thr179, Ala248, Ile315 e Ile368). This result has a correlation with the experimental data found for LASSBio-2070 (**10**), in which the cells treated with this ligand displayed a cell morphology similar to that with microtubule-destabilizing drugs such as CA-4 (**1**), with polymerization inhibition (Figure 4G).

It is also possible to visualize the increase in the number of hydrophobic interactions promoted by the benzyl group with the residues of Val181, Asn256, Met257, Ala314, Ala315, and Lys350. Other fragments of that structure interacted with additional residues important for selectivity of ligands at the colchicine site that act as tubulin destabilizers, such as Asn101, Leu240, Ile316 [32,33]. These results also show that the conformation of LASSBio-2070 (**10**) allowed a similar fitting at the colchicine binding site when compared with CA-4 (**1**). Additionally, LASSBio-2070 (**10**) promoted more interactions than CA-4 (**1**) due to the extra phenyl group that interacts with amino acids within the α-subunit (Asn101, Ala180 and Thr179). This probably justified the molecular reason for LASSBio-2070 (**11**) to act as a destabilizing agent, mimicking CA-4 (**1**) pharmacological behavior as demonstrated in the following docking study.

#### 2.3.2. Docking in the Taxol Binding Site

As shown in Figure 6D, the polar groups of taxol interact in the M-loop through of hydrogen bond between the side chain of Thr276 with the oxetane ring and Arg278 with the carbonyl of benzoacetate group, while the fragment of the molecule containing an amide carbonyl, and a hydroxyl group at C2, makes a hydrogen bond with His229 (H7 loop) and Arg369 (S9 loop), respectively. The same residues (His229 and Arg369) and hydrophobic residues (Val23 and Ala233) that also belong to the S9 loop, make van der Waals interactions with these groups. All these residues are described as important for selectivity of microtubule stabilizing agents (MSAs) [34]. 

LASSBio-1586 (**2**) (Figure 6E) showed a variety of complementary interactions with the M-loop, favored by the anti-periplanar (O=C-N-H) conformation adopted by the amide fragment of the *N*-acylhydrazone subunit [35]. The *N*-acylhydrazone group also acted as a hydrogen acceptor and donor, to interact with Thr276 with both nitrogen atoms. Noteworthy is that the oxetane group of taxol interacts with this residue only as a hydrogen acceptor. Another hydrogen bond was promoted by one of the methoxy groups with Arg278, which are also able to make van der Waals interactions with the residues from H7 loop (Leu217, Leu219, His229 and Leu230). The imine phenyl group interacts with residues from the M-loop (Pro274, Thr276, Gln281 and Leu286) with more intensity in terms of distance when compared with hydrophobic interactions performed by LASSBio-1735 (**3**) (Figure 6F) for the same residues. Additionally, more hydrophobic residues interact with this fragment of the LASSBio-1586 (**2**) on the M-loop. These results possibly contribute to understanding the mechanism by which this molecule acts as a MSA [36,37].

Although LASSBio-1735 (**3**) adopts a different conformation (*sin*-periplanar) related to the amide group (O=C-N-C), its interactions between the trimethoxyphenyl ring and the H7-loop are similar, except for the loss of the hydrogen bond with Arg278, and van der Waals interaction with His229, this loss being counterbalanced by the hydrophobic interaction with Ala233. The main difference occurs when analyzing the interaction at the hydrophobic pocket on the M-loop, in which only the methyl group of the *N*-acylhydrazone subunit is facing towards the cavity, while the phenylmethylidene group is directed to the H7-loop. The change of conformation favors the imine phenyl group to make additional pi-stacking interaction with the Phe272 present on the S9-loop. Together, this result also revealed the capacity of the LASSBio-1735 (**3**) to act as a MSA like LASSBio-1586 (**2**).

#### 2.3.3. Docking in the EGFR

The EGFR structure was selected on the basis of the control compound assayed against the PC-9 cell line expressing EGFR. The structure co-crystallized with erlotinib was retrieved from the Protein Data Bank server (PDB ID: 1M17; resolution: 2.60 Å) [38]. Docking was performed with the GOLD 2022.3.0 program (CCDC). Re-docking of the co-crystallized ligand to identify the most adequate fitness function led to the selection of ChemPLP [38] scoring function. 

**Figure 7:**
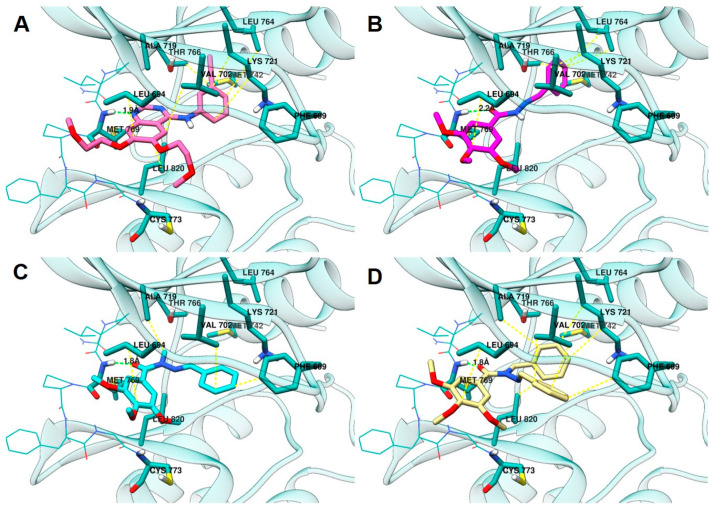
Analysis of the interaction mode in (**A**) erlotinib in pink, (**B**) LASSBio-1586 (**2**) in magenta, (**C**) LASSBio-1735 (**3**) in cyan, and (**D**) LASSBio-2070 (**10**) in yellow, at the ATP binding site (PDB: 1M17). Protein carbon atoms are represented in light blue, oxygen in red, nitrogen in blue and the distances between hydrogen bond are represented by the green line. Hydrophobic interactions are represented by yellow lines.

According to the results, the compounds LASSBio-1586 (**2**) (ChemPLP fitness: 58.3), LASSBio-1735 (**3**) (ChemPLP fitness: 51.7), and LASSBio-2070 (**10**) (ChemPLP fitness: 71.1) fit into the ATP binding site (Figure 7). Like erlotinib (ChemPLP fitness: 70.2; RMSD: 1,4 Å) (Figure 7A), these compounds interact with the hinge region amino acid, Met793, through hydrogen bonding. As shown in Figure 7B,C, the oxygen of the carbonyl group of the *N*-acylhydrazone subunit acts as a hydrogen bond acceptor towards the -NH- group of the Met793 main chain. Additionally, the 3,4,5-trimethoxyphenyl subunit occupies the hydrophobic channel, next to the solvent exposed region, supported by hydrophobic interactions with Leu694. Furthermore, the methoxy groups point toward the solvent in the same manner of the ether groups of erlotinib.

The homologation strategy [10] greatly impacted interaction with the hydrophobic pocket that is occupied by the 3-ethynyl-aniline group of erlotinib (ChemPLP fitness: 70.2; RMSD: 1,4 Å). LASSBio-1586 (**2**) (ChemPLP fitness: 58.3), the non-homologated compound, projects the phenyl group deeply into the hydrophobic pocket, making hydrophobic interactions with residues that interact with the aniline subunit of erlotinib, namely Leu764, Met742, Thr766, and Lys721. 

The methyl homolog **3** (ChemPLP fitness: 51.7) adopts an anti-periplanar conformation (regarding the methyl group and carbonyl oxygen atom) that is in accordance with that previously reported for *N*-methyl-acylhydrazones [39,40]. Consequently, the phenyl group leaves the hydrophobic pocket and moves to the Gly-rich loop, which enables hydrophobic interactions with Phe699 and Val702. 

LASSBio-2070 (**11**) (ChemPLP fitness: 71.1), the benzyl homolog of LASSBio-1586 (**2**), occupies both regions, resulting in higher scoring. It makes hydrophobic interactions with Ala719, Lys721, and Leu820 in the hydrophobic pocked occupied by LASSBio-1586 (**2**), and the aniline subunit of erlotinib. Additionally, LASSBio-2070 (**10**) is also able to interact with Phe699 and Val702 in the Gly-rich loop, comparably to LASSBio-1735 (**3**).

The covalent inhibitor osimertinib [20], which was also biologically evaluated in this work, was covalently docked in this model (Supplementary Material). Due to its ability to occupy all the aforementioned pockets and also to covalently bind to Cys797 of the target, osimertinib had higher scoring (ChemPLP fitness: 123.6; RMSD: 1.6 Å). The non-covalent docking of osimertinib (Supplementary Material) indicated a scoring (ChemPLP fitness: 74.8; RMSD: 1.9 Å) similar to that of erlotinib and LASSBio-2070 (**10**). Studies aiming to develop inhibitors of osimertinib-resistant EGFR highlight that a strong non-covalent complex with the enzyme, in which the inhibitor should occupy multiple pockets of the enzyme, might be favorable to overcome resistance [41,42,43]. 

## 3. Material and Methods

### 3.1. Synthesis and Characterization

All reagents and solvents were purchased from commercial suppliers. The reactions were monitored by thin layer chromatography, which was performed on aluminum sheets pre-coated with silica gel 60 (HF-254, Merck) to a thickness of 0.25 mm. The chromatograms were viewed under ultraviolet light (254–365 nm). For column chromatography Merck silica gel (230–400 mesh) was used. ^1^H NMR, ^13^C NMR and ^13^C DEPT 135 NMR spectra were determined in deuterated dimethyl sulfoxide using a Varian NMR at 400 MHz, 500 MHz and 125 MHz. Chemical shifts are given in parts per million (d) from tretramethylsilane as internal standard and coupling constant values (J) are given in Hertz (Hz). Signal multiplicities are represented by s (singlet), d (doublet), t (triplet), q (quadruplet), m (multiplet) and br (broad signal). All NMR spectra are found in the Supplementary Material.

Infrared (IR) spectra were obtained with an FTIR Thermo Scientific™ Nicolet™ iS10 spectrophotometer in ATR mode using ruby crystal support, and the absorption values were expressed in inverse centimeters (cm^−1^) and the spectra are found in the Supplementary Material. Melting points were determined by Differential Scanning Calorimetry with a Shimadzu’s DSC-60 apparatus up to 300 °C (heating rate: 20 °C/min). DSC-60 equipment calibration were performed using Indium (In) as standard (m.p. 157.2 °C). 

The purity of compounds was determined by HPLC (95%) using the Shimadzu—LC20AD apparatus, a Kromasil 100-5 C18 (4.6 mm × 6250 mm) column and the SPD-M20A detector (Diode Array) at 254 nm for quantification of analyte at a 1 mL/min constant flux. The injector was programmed to inject a volume of 20 µL. The isocratic mobile phases used were CH_3_CN: H_2_O (6:4 *v*/*v*). Mass spectrometry was obtained by positive ionization at BrukerAmaZon SL and data was analyzed in Compass 1.3.SR2 software (Billerica, MA, USA).

#### 3.1.1. Procedure for the Preparation of (*E*)-*N*’-benzylidene-3,4,5-trimetoxybenzohydrazide (**2**; LASSBio-1586)

Compound **2** was prepared following the methodology previously described by Amaral and coworkers and shown in Figure 2. The melting point, ^1^H NMR, ^13^C NMR and IR data agree with previous reports [7]. 

Overall yield: 80%, white solid, m.p. 138–139 °C.

^1^H-NMR (500 MHz, DMSO-d6) δ (ppm): 11.71 (1 H, s, H-8), 8.48 (1 H, s, H-9), 7.73 (2 H, d, J = 5 Hz, H-11/H-15), 7.49–7.45 (3 H, m, H-12/H-13/H-14), 7.24 (2 H, s, H-2/H-6), 3.87 (6 H, s, H-16/H-18), 3.73 (3 H, s, H-17).

^13^C-NMR (125 MHz, DMSO-d6) δ (ppm): 163.0 (C-7), 153.1 (C-3/C-5), 148.2 (C-9), 140.9 (C-4), 134.7 (C-10), 130.5 (C-13), 129.3 (C-11/C-15), 128.9 (C-1), 127.5 (C-12, C-14), 105.6 (C-2/C-6), 60.5 (C-17), 56.5 (C-16/C-18)

I.R. (ATR- FTIR, cm^−1^): 3181 (N-H), 2995–2839 (CH3, CH2, CH), 1646 (-C=O), 1624–1452 (C=C), 1584 (C-N), 1118 (C-O).

99.4% purity in HPLC (R.T. = 9,63; CH_3_CN:H_2_O (6:4 *v*/*v*)). MS: m/z = 315.1 (M+H)^+^. 

#### 3.1.2. General Procedure for the Preparation of the *N*-Alkylated Acylhydrazones (**3**–**12**)

A solution of LASSBio-1586 (**2**) (0.4 g, 1.27 mmol) in 7 mL of acetone was added to 3.82 mmol of sodium carbonate. The resultant suspension was stirred at room temperature for 50 min. Then, the appropriate alkyl halide or benzyl bromide (0.48 mL, 7.63 mmol) was added to the suspension and the reaction mixture was heated for 24 h at 40 °C. The acetone was removed under reduced pressure and the resulting material was suspended in 2 mL of ethanol, filtered, and washed with petroleum ether. Recrystallization of the target products was performed using a mixture of ethanol/water. Yields and characterization pattern are described below: 

(*E*)-*N*’-benzylidene-3,4,5-trimethoxy-*N*-methylbenzohydrazide (**3**; LASSBio-1735). 

Yields: 71%, White solid; m.p.110–110 °C (in agreement with Amaral et al., 2014). 

^1^H-NMR (500 MHz, DMSO-d6) δ (ppm): 8.04 (1 H, s, H-9), 7.59–7.57 (2H, m, H11/H15), 7.42–7.34 (3 H, m, H12/H13/H14), 7.00 (2 H, s, H2/H6), 3.77 (6 H, s, H16/H18), 3.75 (3 H, s, H17), 3.50 (3 H, s, H8).

^13^C-NMR (125 MHz, DMSO-d6) δ (ppm): 169.5 (C-7), 152.2 (C-3/C-5), 140.8 (C-9), 139.5 (C-4), 135.3 (C-10), 130.8 (C-13), 129.9 (C-1), 129.2 (C-11/C-15), 127.2 (C-12/C-14), 108.0 (C-2/C-6), 60.6 (C-17), 56.3 (C-16/C-18), 29.4 (C-8).

IR (ATR- FTIR, cm^−1^): 2921–2832 (CH3, CH2, CH), 1641 (-C=O), 1580 (C-N), 1503–1453 (C=C), 1131 (C-O).

99.8% purity in HPLC (R.T. = 9.15 min; CH_3_CN:H_2_O (6:4 *v*/*v*)). MS: m/z = 329.1 (M+H)^+^.

(*E*)-*N*’-benzylidene-*N*-ethyl-3,4,5-trimethoxybenzohydrazide (**4**; LASSBio-2071).

Yields: 60%, White solid; m.p.83–84 °C;

^1^H-NMR (500 MHz, DMSO) δ (ppm): 8.10 (1H, s, H-10), 7.60–7.58 (2H, m, H-12/H-16), 7.42–7.34 (3H, m, H-13/H-14/H-15), 6.98 (2H, s, H-2/H-6), 4.18 (2H, q, J = 7.0 Hz, H-8), 3.77 (6H, s, H-17/H-19), 3.75 (3H, s, H-18), 1.20 (3H, t, J = 7.0, H-9).

^13^C NMR (125 MHz, DMSO) δ (ppm): 169.2 (C-7), 152.2 (C-3, C-5), 140.3 (C-10), 139.6 (C-4),135.4 (C-11), 131.0 (C-14), 129.9 (C-1), 129.1 (C-12, C-16), 127.3 (C-13, C-15), 108.0 (C-6, C-2), 60.6 (C-18), 56.4 (C-17, C-19), 36.3 (C-8), 11.3 (C-9)

^13^C DEPT-135 NMR (126 MHz, DMSO) δ (ppm): 140.43 (s), 129.98 (s), 129.19 (s), 127.30 (s), 108.08 (s), 60.66 (s), 11.38 (s).

IR (ATR- FTIR, cm^−1^): 2968–2833 (CH3, CH2, CH), 1633 (-C=O), 1579 (C-N), 1510–1417 (C=C), 1125 (C-O).

99.1% purity in HPLC (R.T. = 9.27 min; CH_3_CN:H_2_O (6:4 *v*/*v*)). MS: m/z = 342.9 (M+H)^+^.

(*E*)-*N*’-benzylidene-3,4,5-trimethoxy-*N*-propylbenzohydrazide (**5**; LASSBio-2118).

Yields: 30%, Yellow oil;

^1^H-NMR (500 MHz, DMSO) δ (ppm): 8.09 (1H, s, H-11), 7.59–7.57 (2H m, H-13/H-17), 7.41–7.31 (3H, m, H-14/H-15/H-16,), 6.96 (2H, s, H-2/H-6), 4.09 (2H, t, *J* = 7.2 Hz, H-8), 3.77 (6H, s, H-18/H-20), 3.74 (3H, s, H-19), 1.66–1.60 (2H, m, H-9), 0.97 (3H, t, *J* = 7.3 Hz, H-10).

^13^C-NMR (125 MHz, DMSO) δ (ppm): 169.6 (C-7), 152.2 (C-3/C-5), 140.4 (C-9), 139.5 (C-4), 135.4 (C-12), 131.1 (C-15), 130.0 (C-1), 129.1 (C-13/C-17), 127.3 (C-14, C-16), 107.9 (C-6, C-2), 60.6 (C-19), 56.3 (C-18, C-20), 42.7 (C-8), 19.0 (C-9, s), 11.67 (C-10).

^13^C DEPT-135 NMR (126 MHz, DMSO) δ (ppm): 140.43 (s), 130.00 (s), 129.19 (s), 127.30 (s), 107.95 (s), 60.65 (s), 11.68 (s).

IR (ATR- FTIR, cm^−1^): 3050 (NH), 2940–2800 (CH3, CH2, CH), 1648,3 (-C=O), 1581–1449 (C=C), 1123 (C-O).

98% purity in HPLC (R.T. = 9.55 min; CH_3_CN:H_2_O (6:4 *v*/*v*)). MS: m/z = 379.1 (M+H)^+^.

(*E*)-*N*’-benzylidene-*N*-butyl-3,4,5-trimethoxybenzohydrazide (**6**; LASSBio-2121).

Yields: 30%, Yellow oil;

^1^H-NMR (500 MHz, DMSO) δ (ppm): 8.09 (1H, s, H-12), 7.59–7.56 (2H, m, H-14/H-18), 7.41–7.34 (3H, m, H-15/H-16/H-17), 6.96 (2H, s, H-2/H-6), 4.12 (2H, t, *J* = 7.4 Hz, H-8), 3.77 (6H, s, H-19/H-21), 3.74 (3H, s, H-20), 1.63–1.57 (2H, m, H-9), 1.44–1.22 (2H, m, H-10), 0.95 (3H, t, *J* = 7.3 Hz, H-11).

^13^C-NMR (125 MHz, DMSO) δ (ppm): 169.5 (C-7), 152.2 (C-3/C-5), 140.3 (C-12), 139.6 (C-4), 135.4 (C-13), 131.1 (C-16), 130.0 (C-1), 129.1 (C-14/C-18), 127.3 (C-15/C-17), 108.0 (C-2/C-6), 60.6 (C-20), 56.4 (C-19/C-21), 40.9 (C-8), 27.8 (C-9), 20.1 (C-10), 14.2 (C-11).

^13^C DEPT-135 NMR (126 MHz, DMSO) δ (ppm): 140.37 (s), 130.01 (s), 129.19 (s), 127.32 (s), 108.00 (s), 60.66 (s), 56.41 (s), 14.22 (s).

IR (ATR- FTIR, cm^−1^): 3050 (NH), 2940–2800 (CH_3_, CH_2_, CH), 1648,3 (-C=O), 1581–1449 (C=C), 1123 (C-O). 

99.4% purity in HPLC (R.T. = 10.56 min; CH_3_CN:H_2_O (6:4 *v*/*v*)). MS: m/z = 371.1 (M+H)^+^.

(*E*)-*N*’-benzylidene-*N*-(sec-butyl)-3,4,5-trimethoxybenzohydrazide (**7**; LASSBio-2119)

Yields: 60%, Yellow oil;

^1^H-NMR (500 MHz, DMSO) δ (ppm): 8.45 (1H, s, H-12), 7.75–7.73 (2H, m, H-14/H-16), 7.49–7.43 (3H, m, H-15/H-16/H-17), 7.05 (2H, s, H-2/H-6), 5.13–5.07 (1H, m, H-8), 3.76 (6H, s, H-19/H-21), 3.73 (3H, s, H-20), 1.80–1.67 (2H, m, H-10,), 1.36 (3H, d, *J* = 5.5 Hz, H-9), 0.97 (3H, t, *J* = 7.4 Hz, H-11).

^13^C-NMR (125 MHz, DMSO) δ (ppm): 162. 9 (C-7), 157.0 (C-3,C-5), 152.4 (C-12), 139.9 (C-4), 135.2 (C-13), 130.7 (C-16), 129.2 (C-14, C-18), 128.0 (C-15,C-17), 126.5 (C-1), 108.3 (C-2/C-6), 74.1 (C-8), 60.6 (C-20), 56.3 (C-19, C-21), 28.7 (C-10), 19.4 (C-9), 10.12 (H-11).

^13^C NMR DEPT 135 (126 MHz, DMSO) δ 157.06 (s), 130.78 (s), 128.29 (s), 128.03 (s), 108.30 (s), 74.16 (s), 60.63 (s), 56.34 (s), 19.42 (s), 10.13 (s).

IR (ATR- FTIR, cm^−1^): 3050 (NH), 2968–2835 (CH_3_, CH_2_, CH), 1640.2 (-C=O), 1576–1462 (C=C), 1123 (C-O).

99.4% purity in HPLC (R.T. = 12.40 min; CH_3_CN:H_2_O (6:4 *v*/*v*)). MS: m/z= 371.30 (M+H)^+^. 

(*E*)-*N*-allyl-*N*’-benzylidene-3,4,5-trimethoxybenzohydrazide (**8**; LASSBio-2069).

Yields: 71%, White solid; m.p.64–65 °C;

^1^H-NMR (400 MHz, DMSO) δ(ppm): 7.95 (1H, s, H-11), 7.55 (2H, H-13, H-18, d, *J* = 5 Hz), 7.42–7.35 (3H, m, H-15/H-16/H-17), 7.03 (2H, s, H-2/H-6), 5.95–5.87 (1H, m, H-9), 5.23 (1H, dd, *J* = 10 Hz), 5.18 (1H, dd, *J* = 15 Hz, H-10′), 4.81 (2H, d, *J* = 5 Hz, H-8), 3.79 (6H, s, H-18/H-20), 3.76 (3H, s, H-19).

^13^C-NMR (100 MHz, DMSO) δ (ppm): 169.4 (C-13), 152.3 (C-17), 135.1 (C-1), 131.3 (C-3), 130.6 (C-2), 130.1 (C-18), 129.2 (C-25), 127.3 (C-21), 117.2 (C-26), 108.1 (C-4/C-6), 60.6 (C-11), 56.4 (C-10/C12), 43.91 (C-24).

IR (ATR- FTIR, cm^−1^): 3090 (NH), 2997–2831 (CH3, CH2, CH), 1650.8 (-C=O), 1636–1452 (C=C), 1122 (C-O).

98.3% purity in HPLC (R.T. = 6.37 min; CH_3_CN:H_2_O (6:4 *v*/*v*)). MS: m/z = 355.2 (M+H)^+^. 

(*E*)-*N*’-benzylidene-3,4,5-trimethoxy-*N*-(prop-2-yn-1-yl)benzohydrazide (**9**; LASSBio-2072).

Yields: 70%, White solid; m.p.133–134 °C;

^1^H-NMR (500 MHz, DMSO) δ (ppm): 8.14 (1H, s, H11), 7.61–7.58 (2H, m, H-13/H-17), 7.45–7.38 (3H, m, H-14/H-15/H-16), 7.04 (2H, s, H-2/H-6), 4.99 (2H, d, J = 5.0 Hz, H-8), 3.79 (6H, s, H-18/H-20), 3.76 (3H, s, H-19).

^13^C-NMR (125 MHz, DMSO) δ (ppm): 168.9 (C-7), 152.3 (C-3, C-5), 141.8 (C-11), 140.0 (C-4), 134.8 (C-12), 130.3 (C-15), 129.8 (C-12), 129.3 (C-17), 127.3 (C-14, C-16), 108.1 (C-6, C-2), 77.9 (C-9), 75.7 (C-10), 60.6 (C-19), 56.4 (C-18, C20), 31.5 (C-10)

IR (ATR- FTIR, cm^−1^): 3242 (C-H), 2935–2834 (CH3), 1641(C=O), 1583 (C-N), 1126 (C-O).

97.6% purity in HPLC (R.T. = 8.83 min; CH_3_CN:H_2_O (6:4 *v*/*v*)). MS: m/z = 352.9 (M+H)^+^. 

(*E*)-*N*-benzyl-*N*’-benzylidene-3,4,5-trimethoxybenzohydrazide (**10**; LASSBio-2070).

Yields: 46%, White solid; m.p.130–131 °C;

1H-NMR (400 MHz, DMSO) δ (ppm): 7.9 (1H, s, H-15), 7.48–7.46 (2H, m, H-17, H-21), 7.40–7.33 (7H, m, H-11, H-12, H-13, H-18, H-19, H-20, H-10), 7.30–7.26 (1H, s, H-14), 7.10 (2H, s, H-6, H-2), 5.44 (2H, s, H-8), 3.81 (6H, s, H-22, H-24), 3.77 (3H, s, H-23).

^13^C-NMR (100 MHz, DMSO) δ (ppm): 170.0 (C-7), 152.3 (C-2/C-5), 141.1 (C-15), 139.9 (C-4), 135.8 (C-16), 134.9 (C-9), 130.5 (C-19), 130.1 (C-12), 129.2 (C-17, C-21, C-11, C-13), 127.6 (C-1), 127.2 (C-18, C-20, C-10, C-14)), 108.3 (C-2/C-6), 60.6 (C-23), 56.5 (C-22/C-24), 45.0 (C-8).

^13^C NMR DEPT 135 (126 MHz, DMSO) δ 141.17 (s), 130.19 (s), 128.25 (s), 127.29 (s), 127.27(s),108.30 (s), 60.70 (s), 56.50 (s). 

IR (ATR- FTIR, cm^−1^): 3090 (NH), 3000–2830 (CH3, CH2, CH), 1635.8 (-C=O), 1607–1454 (C=C), 1125 (C-O).

98.4% purity in HPLC (R.T. = 6.38 min; CH_3_CN:H_2_O (6:4 *v*/*v*)). MS: m/z = 405.2 (M+H)^+^. 

Ethyl (*E*)-*N*-(benzylideneamino)-*N*-(3,4,5-trimethoxybenzoyl)glycinate (**11**; LASSBio-2074).

Yields: 65%, White solid; m.p.91–92 °C;

^1^H-NMR (500 MHz, DMSO) δ (ppm): 7.99 (1H, s, H-12), 7.57–7.55 (2H, d, *J* = 8.0, H-14/H-18), 7.46–7.37 (3H, m, H-15/H-16/H-17), 7.04 (2H, s, H-2/H-6,), 5.00 (2H, s, H-8), 4.22–4.17 (2H, q, H-10), 3.78 (6H, s, H-19/H-21), 3.76 (3H, s, H-20), 1.24 (3H, t, *J* = 7.1 Hz, H-11).

^13^C-NMR (125 MHz, DMSO) δ (ppm): 169.5 (C-9), 167.7 (C-7),152.3 (C-5/C-3), 141.1 (C-12), 140.0 (C-4), 134.9 (C-13), 130.2 (C-1), 129.8 (C-16), 129.3 (C-14/C-18), 127.3 (C-15/C-17), 108.1 (C-2/C6), 61.6 (C-8), 60.6 (C-20), 56.4 (C-19/C-21), 43.8 (C-10), 14.5 (C-11).

^13^C DEPT-135 NMR (126 MHz, DMSO) δ 141.16 (s), 130.31 (s), 129.31 (s), 128.21 (s), 127.38 (s), 108.17 (s), 60.68 (s), 56.40 (s), 40.18 (s), 14.51 (s).

IR (ATR- FTIR, cm^−1^): 3000 (NH), 2941–2838 (CH3, CH2, CH), 1787,8 (-C=O), 1664,2 (-C=O), 1125 (C-O).

98.0% purity in HPLC (R.T. = 9.69 min; CH_3_CN:H_2_O (6:4 *v*/*v*)). MS: m/z = 401.0 (M+H)^+^.

(*E*)-*N*’-benzylidene-3,4,5-trimethoxy-*N*-(2-morpholinoethyl)benzohydrazide (**12**; LASSBio-2122).

Yields: 36%, White solid; m.p.91–92 °C;

^1^H-NMR (500 MHz, DMSO) δ (ppm): 8.12 (1H, s, H-14), 7.58–7.55 (2H, m, H-15/H-20), 7.42–7.34 (3H, m, H-17/H-18/H-19), 6.96 (2H, s, H-2/H-6), 4.26 (2H, t, *J* = 6.9 Hz, H-8), 3.77 (6H, s, H-21/H-23), 3.75 (3H, s, H-22), 3.57 (4H, t, *J* = 5 Hz, H-12, H-13), 2.57 (2H, t, *J* = 5 Hz, H-9), 2.5 (4H, m, H-10/H-11).

^13^C-NMR (125 MHz, DMSO) δ (ppm): 169.6 (C-7), 152.2 (C-5/C-3), 140.6 (C-14), 139.6 (C-4), 135.3 (C-15), 131.0 (C-1), 130.0 (C-18), 129.2 (C-20/C-16), 127.3 (C-19/C17), 107.9 (C-2/C-6), 66.7 (C-12/C13), 60.6 (C-22), 56.4 (C-10/C-11/C-21/C-23), 54.1 (C-8), 53.9 (C-9).

^13^C DEPT-135 NMR (126 MHz, DMSO) δ (ppm): 140.66 (s), 130.05 (s), 129.21 (s), 127.35 (s), 107.96 (s), 60.66 (s), 56.40 (s).

IR (ATR- FTIR, cm^−1^): 3000 (NH), 2958–2843 (CH3, CH2, CH), 1660.8 (-C=O), 1608–1462 (C=C), 1127 (C-O).

98.4% purity in HPLC (R.T. = 9.27 min; CH_3_CN:H_2_O (6:4 *v*/*v*)). MS: m/z = 428.2 (M+H)^+^.

### 3.2. Cell Culture

The target compounds were tested (0.0001–100 µM) for cytotoxic activity against selected human cancer cell lines, such as HL-60 (acute promyelocytic leukemia), MCF-7 (mammary adenocarcinoma), PC-3 (prostate adenocarcinoma), H1975 (non-small cell lung cancer—EGFR^L858R+T790M^), PC-9 (non-small cell lung cancer—EGFR^L858R^) H292 (mucoepidermoid pulmonary carcinoma—EGFR wild type), LoVo (colorectal adenocarcinoma—EGFR_wild type_) and GM16000 (lymphocyte). All the human cancer cell lines were purchased from Cell Bank of Rio de Janeiro—BCRJ. They were cultured in RPMI 1640 medium with 10% (*v*/*v*) FBS, 2 mM glutamine, at 37 °C in a humidified incubator containing 5% CO_2_. 

### 3.3. Cell Viability Assay

The cytotoxicity of the compounds was evaluated by the MTT assay. The cell lines were cultured in 96 well plates for 72 h with a density of 0.1 × 10^5^ cells/mL to 0.7 × 10^5^ cells/mL to adherent cell lines. For lymphocytic lines, a density of 0.3 × 10^6^ cells/mL was used. Afterwards they were treated with varying concentrations of the target compounds 2–12 (0.0001–100 µM), CA-4 (**1**) and pelitinib at 37 °C, in triplicate. The medium was incubated with 20 μL of 5 mg/mL MTT solution for 3 h in a humidified incubator containing 5% CO_2_ [11].

The purple-colored formazan crystals formed in the wells were dissolved in DMSO and their absorbance was measured at 595 nm with a microplate reader (SpectraMax M5, Molecular Devices). The CC_50_ values were calculated from the means (95%, confidence interval (CI)), using GraphPad Prism^®^, version 7.

### 3.4. Flow Cytometry Analysis

Flow cytometry was applied to help elucidate the mechanism of action of the studied compounds, both to verify the cell cycle phase and to verify the action on EGFR protein kinase. CA-4 (**1**) (0.04 µM) was used as a control for the cell cycle assay and the compounds were tested using a concentration equivalent to CC_50_ value of each compound determined in the MTT assay of 72 h for cell cycle kit: LASSBio-1586 (**2**) (2.0 µM), LASSBio-1735 (**3**) (0.08 µM), LASSBio-2070 (**10**) (3.0 µM) and LASSBio-2074 (**11**) (1.5 µM. For the EGFR assay osimertinib (0.05 µM) and erlotinib (0.1 µM) were used as standards, with LASSBio-1586 (**2**) (0.06 µM), and the compounds LASSBio-1735 (**3**) (0.02 µM) and LASSBio-2070 (**10**) (0.03 µM). 

The anti-tubulin effect was evaluated by seeding H1975, H292 and PC-9 into 6-well plates at a density of 2 × 10^5^ per well. The cells were treated with the selected compounds for 72 h. Cells were harvested and stained according to the protocols of The Guava Muse^®^ Cell Cycle Kit (MCH100106) to quantitatively measure the percentage of cells in the G2/M phases of the cell cycle and the EGFR-RTK Activation Dual Detection Kit (MCH200102) (Luminex^®^) to measure EGFR phosphorylation in relation to total EGFR expression in the cell population, according to the manufacturer’s instructions for use in test kits using a Guava Muse^®^ Cell Analyzer (Luminex^®^). Each experiment was performed at least three times independently [44,45].

The evaluation of EGFR activation and phosphorylation was performed by the Guava Muse^®^ EGFR-RTK Activation Dual Detection Kit (catalog number MCH200102, Luminex, (Austin, TX, USA) containing the anti-EGFR-PECy5 antibody for measuring total EGFR and the anti-phospho-EGFR antibody (Tyr1173)—Alexa Fluor^®^ 555 for measuring EGFR, activated and performed according to the manufacturer’s instructions. The cells were trypsinized, centrifuged at 300× *g* for 5 min, and, after the labeling protocol, the samples were taken for reading in the Guava Muse^®^ Cell Analyzer. In this test the compounds were tested and used at concentrations 10 times lower than the CC_50_ in 72 h.

### 3.5. Immunofluorescence and Digital Image Acquisition

The cell line H1975 was chosen for this experiment, since the size of the cells would be best to allow the most effective visualization of the effects of the compounds in the microtubules, which were labeled together with the cell nucleus. Cells were plated at a concentration of 5 × 10^5^ cells/well and treated for 48 h with compounds CA-4 (**1**) (0.3 µM), Taxol (0.1 µM), Vincristine (0.5 µM), LASSBio-1586 (**2**) (10 µM), LASSBio-1735 (**3**) (0.5 µM), LASSBio-2070 (**10**) (4.5 µM); and LASSBio-2074 (**11**) (20 µM), at concentrations of 5 times the 48 h CC_50_. After incubation, cells were rinsed with PBS and fixed with 4% paraformaldehyde in PBS for 10 min at room temperature. They were then permeabilized with 0.5% Triton-X 100 in PBS 3 times for 10 min. The same solution was used for all subsequent washing steps. Cells were incubated with an anti-beta-tubulin antibody for 1 h at 37 °C. After incubation, cells were washed for 30 min and incubated with Alexa Fluor^®^ 488, conjugated secondary antibody for 1 h at 37 °C, and nuclei were labeled with DAPI (0.1 mg/mL in 0.9% NaCl) [46].

Live cultured cells grown in coverslips were mounted in Prolong Gold solution (Molecular Probes, USA) and were imaged on a Leica TCS SPE confocal microscope (Leica Microsystems, Germany) using 63x 1.3 N.A. oil immersion and the images acquired by the LAS X software. Cell morphology, focusing on the microtubules was analyzed, qualitatively, using the public domain NIH ImageJ Program (developed at the National Institutes of the USA Health and available on the internet at http://imagej.nih.gov/ij/ (accessed on 1 September 2021).

### 3.6. Molecular Docking Studies

All compounds were drawn with Spartan 18′ (Wavefunction Inc., Irvine, CA, USA), and the lowest energy tautomer was energy-minimized at the semi-empirical PM6 level. Protonation state of docked compounds was determined considering the major species at pH 7.4, using ACD/Percepta program. The EGFR structure co-crystallized with erlotinib was retrieved from the Protein Data Bank server (PDB ID: 1M17) [47]. The tubulin structure co-crystallized with colchicine and taxol was retrieved from the Protein Data Bank server (PDB ID: 5XIW and 6WVR) [31,34]. The selected proteins were prepared by adding hydrogen atoms and adjusting the protonation states of amino acids (Dock Prep, UCSF Chimera). The set of amino acid residues selected as the binding site was determined within 6 Å distance from co-crystallized ligand in the ATP biding site, and crystallographic waters were removed during the docking runs. Docking was performed with the GOLD 2022.3.0 program (CCDC). Re-docking of the co-crystallized ligand to identify the most adequate fitness function led to the selection of ChemPLP for both target (EGFR and tubulin) [39] scoring function with the lowest root-mean square deviation (RMSD). For the osimertinib RMSD calculation, a superposition of its co-crystal structure (PDB ID: 6JX0; resolution of 2.53 Å) [48] with the structure used for docking was carried out before docking osimertinib to the erlotinib co-crystallized protein structure. For each compound, 100 docking runs were carried out using very flexible genetic algorithm settings (GOLD 2022.3.0, CCDC). Docking with tubulin was carried out with default settings (genetic algorithm with 100% of flexibility). Amino acid residues were considered rigid during the calculation for both proteins. Figures were produced using UCSF Chimera.

## 4. Conclusions

To the best of our knowledge our work describes, for the first time, the identification by phenotypic models of a compound of low structural complexity that acts by modulating, simultaneously, microtubules and EGFR activities. The benzyl homolog **10** (LASSBio-2017) is suggested as a putative dual inhibitor of tubulin and EGFR, displaying good cytotoxic potency in different human tumor cell lines. The mode of interaction with the target proteins was studied by molecular docking and the information will used in a further lead-optimization step. 

## Figures and Tables

**Table 1:** Overall yields, physical aspect, melting point, purity by HPLC and retention time of compounds **2**–**12**.

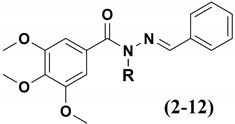						
Compounds	R	Overall Yields (%)	Physical Appearance	Melting Point (°C)	Determined Purity (%)	Retention Time in HPLC (min)
LASSBio-1586 (**2**)	H	70	white powder	138.33	99.94	9.63
LASSBio-1735 (**3**)	Methyl	71	white powder	110.56	99.90	9.15
LASSBio-2071 (**4**)	Ethyl	60	white powder	83.94	99.14	9.27
LASSBio-2118 (**5**)	*N*-propyl	28	yellow oil	-	98.04	9.55
LASSBio-2121 (**6**)	*N*-butyl	26	yellow oil	-	99.4	10.86
LASSBio-2119 (**7**)	Sec-butyl	60	yellow oil	-	99.8	12.40
LASSBio-2069 (**8**)	Allyl	71	white powder	64.47	98.23	6.37
LASSBio-2072 (**9**)	Propynyl	70	white powder	133.91	97.61	8.83
LASSBio-2070 (**10**)	Benzyl	46	white powder	130.12	98.33	6.38
LASSBio-2074 (**11**)	Methylene Carbethoxy	65	white powder	91.67	98.00	9.69
LASSBio-2122 (**12**)	Morpholine Ethyl	36	white powder	147.64	98.34	9.27

**Table 2:** Cytotoxic potency activity (mean CC_50_ ± CI) of compounds **2**–**12**, and the standards CA-4 and pelitinib, against tumor cell lines and GM16000 at 72 h.

Compounds	CC_50_ (µM) (CI)							
	HL-60	H1975	H292	LoVo	MCF-7	PC-3	PC-9	GM16000
**CA-4 (1)**	0.077 (0.029–0.201)	0.035 (0.016–0.079)	0.013 (0.006–0.02)	0.039 (0.015–0.079)	8.58 (5.407- 10.61)	0.096 (0.072–0.2829)	0.01 (0.008–0.020)	0.064 (0.023–0.176)
**Pelitinib**	2.079 (1.246–3.468)	0.512 (0.433–0.605)	0.07835 (0.05–0.012)	0.037 (0.014–0.069)	3.91 (3.103–4.927)	2.485 (1.809–3.413)	0.0591 (0.037–0.069)	2.749 (2.223–3.399)
**LASSBio-1586 (2)**	2.127 (1.784–2.535)	1.568 (1.271–1.935)	1.398 (1.048–1.864)	0.039 (0.015–0.069)	7.53 (5.217–10.87)	1.619 (1.295–2.023)	0.587 (0.512–0.672)	1.507 (1.103–2.058)
**LASSBio-1735 (3)**	0.039 (0.023–0.065)	0.079 (0.043–0.143)	0.250 (0.174–0.357)	0.149 (0.092–0.243)	0.276 (0.137–0.557)	0.201 (0.117–0.346)	0.090 (0.045- 0.179)	0.131 (0.068–0.254)
**LASSBio-2071 (4)**	0.231 (0.113- 0.475)	4.861 (1.504–5.721)	2.546 (1.443–4.491)	0.301 (0.162–0.559)	1.238 (0.266–5.756)	1.660 (1.248–2.207)	0.384 (0.205–0.718)	5.127 (2.344–11.21)
**LASSBio-2118 (5)**	0.301 (0.190–0.478)	0.775 (0.397–1.512)	3.722 (2.177–6.364)	0.031 (0.005–0.192)	0.144 (0.030–0.670)	0.432 (0.250–0.746)	0.136 (0.071- 0.258)	0.843 (0.391–1.818)
**LASSBio-2121 (6)**	2.171 (1.678–2.809)	2.935 (1.035–4.323)	9.553 (6.323–14.43)	0.5 (0.256–0.969)	0.500 (0.187–1.333)	0.696 (0.445–1.088)	0.096 (0.050–0.184)	0.961 (0.385–2.397)
**LASSBio-2119 (7)**	ND *	ND *	ND *	ND *	ND *	ND *	ND *	ND *
**LASSBio-2069 (8)**	1.348 (0.956–1.901)	3.739 (1.948–4.741)	0.778 (0.440–1.378)	0.298 (0.137–0.650)	1.596 (0.589–4.324)	0.462 (0.240–0.889)	0.387 (0.224–0.667)	0.830 (0.317–2.174)
**LASSBio-2072 (9)**	0.158 (0.088–0.283)	3.979 (1.195–5.253)	3.144 (1.825–5.416)	0.399 (0.202–0.786)	2.318 (1.297–8.07)	2.250 (1.556–3.252)	0.460 (0.250–0.846)	0.612 (0.329–1.138)
**LASSBio-2070 (10)**	0.172 (0.092- 0.322)	2.791 (1.374–4.81)	0.883 (0.395–1.974)	0.208 (0.100–0.431)	1.7 (0.586–4.932)	0.361 (0.171–0.761)	0.337 (0.173–0.654)	1.046 (0.498–2.196)
**LASSBio-2074 (11)**	0.126 (0.061–0.262)	1.204 (0.602–4.013)	0.7503 (0.325–1.729)	0.106 (0.039–0.285)	0.869 (0.175–2.305)	1.361 (0.772–2.397)	0.202 (0.112–0.364)	4.194 (1.767–9.955)
**LASSBio-2122 (12)**	4.150 (2.761–6.238)	2.482 (1.574–4.881)	2.791 (1.362–5.719)	0.421 (0.225–0.787)	4.114 (2.546–6.649)	2.396 (1.385–4.146)	0.871 (0.496–1.528)	5.649 (4.905–6.505)

* ND: means not determined; CI: Confidence interval; CC_50:_ Cytotoxic concentration of the extracts to cause death to 50% of viable cells in vitro.

**Table 3:** Cytotoxic selectivity index (SI) of CA-4, pelitinib, LASSBio-1586 and its homologs **3**–**12** at 72 h.

Compounds	SI						
	GM16000/ HL-60	GM16000/ H1975	GM16000/ H292	GM16000/ LoVo	GM16000/ MCF-7	GM16000/ PC-3	GM16000/ PC-9
**CA-4 (1)**	0.8	1.8	4.5	1.6	0.007	0.7	6.4
**Pelitinib**	1.3	5.4	35.3	74.3	0.7	1.1	46.5
**LASSBio-1586 (2)**	0.7	1.0	1.0	**39.0**	0.2	0.9	2.6
**LASSBio-1735 (3)**	3.4	1.7	0.5	0.9	0.5	0.6	1.4
**LASSBio-2071 (4)**	**22.2**	1.0	2.0	**17.0**	4.1	3.0	**13.3**
**LASSBio-2118 (5)**	2.8	1.1	0.2	**27.2**	5.9	1.9	6.2
**LASSBio-2121 (6)**	0.4	0.3	0.1	2.0	1.9	1.4	**10.0**
**LASSBio-2069 (8)**	0.6	0.2	1.0	2.8	0.5	1.8	2.1
**LASSBio-2072 (9)**	4.0	0.2	0.2	1.5	0.3	0.3	1.3
**LASSBio-2070 (10)**	6.0	0.4	1.2	5.0	0.6	2.9	3.1
**LASSBio-2074 (11)**	**33.3**	3.5	5.6	**39.5**	4.8	3.0	**20.8**
**LASSBio-2122 (12)**	1.4	2.3	2.0	**13.4**	1.4	2.3	6.5

**Table 4:** Cytotoxic potency activity (mean CC_50_ ± CI) of compounds **3**–**12**, and the standards CA-4, pelitinib and LASSBio-1586 (**2**), against tumor cell lines and GM16000 at 48 h.

Compounds	CC_50_ (µM) (CI)					
	HL-60	H1975	H292	LoVo	PC-9	GM16000
**CA-4 (1)**	1.299 (0.639–2.635)	0.058 (0.024–0.140)	0.091 (0.057–0.143)	0.1795 (0.073–0.439)	0.025 (0.011–0.055)	0.016 (0.004–0.07)
**Pelitinib**	3.419 (2.938–3.980)	2.569 (1.993–3.311)	0.480 (0.343–0.731)	1.697 (1.386–2.078)	0.325 (0.258–0.411)	0.899 (0.6–1.35)
**LASSBio-1586 (2)**	3.592 (3.099–4.163)	2.017 (0.054–0.171)	2.186 (1.666–2.870)	1.720 (1.266–2.338)	0.940 (0.761–1.163)	0.763 (0.48–1.215)
**LASSBio-1735 (3)**	8.768 (7.584–10.14)	0.096 (1.600–2.542)	0.152 (0.082–0.281)	0.479 (0.273–0.841)	1.341 (1.032–1.744)	0.059 (0.019–0.181)
**LASSBio-2071 (4)**	6.033 (4.064–8.957)	4.905 (3.573–6.732)	0.300 (0.191–0.980)	3.052 (1.547–6.019)	0.881 (0.544–1.427)	1.996 (1.134–3.513)
**LASSBio-2118 (5)**	1.457 (0.759–2.796)	1.124 (0.557–2.266)	0.181 (0.052–0.629)	0.740 (0.209–2.622)	0.263 (0.133–0.52)	1.096 (0.60–2.00)
**LASSBio-2121 (6)**	2.606 (1.605–4.231)	1.713 (1.041–2.818)	0.215 (0.081–0.567)	2.004 (0.832–4.823)	0.327 (0.173–0.618)	1.196 (0.623–2.296)
**LASSBio-2069 (8)**	0.526 (0.272–1.019)	1.650 (0.813–3.349)	0.154 (0.062–0.385)	0.808 (0.278–2.348)	0.276 (0.156–0.490)	0.095 (0.404–2.963)
**LASSBio-2072 (9)**	5.848 (4.131–8.278)	5.342 (3.556–8.027)	0.450 (0.176–1.150)	3.052 (1.547–6.019)	1.273 (0.669–2.420)	2.524 (1.697–3.754)
**LASSBio-2070 (10)**	6.291 (5.159–7.673)	0.903 (0.512–1.594)	0.147 (0.056–0.383)	2.125 (0.954–4.734)	0.244 (0.135–0.443)	0.935 (0.442–1.974)
**LASSBio-2074 (11)**	5.688 (3.618–8.945)	4.827 (2.988–7.799)	0.593 (0.199–1.769)	2.639 (1.160–4.003)	1.405 (0.636–3.098)	3.911 (1.774–6.21)
**LASSBio-2122 (12)**	5.094 (3.643–7.122)	6.367 (4.708–8.609)	0.412 (0.159–1.068)	1.052 (0.450–2.455)	1.499 (0.928–2.421)	3.8 (1.658–5.52)

CI: Confidence interval. CC_50_: Cytotoxic concentration of the extracts to cause death to 50% of viable cells in vitro.

## Data Availability

Data is contained within the article and Supplementary Materials.

## Supplementary Materials

The following are available online at https://www.mdpi.com/article/10.3390/ph15080913/s1, NMR and IR Spectrum data, selectivity Index (SI) data at 48h of compounds and covalent and non-covalent docking of Osimertinib overlaid with LASSBio-1586 (2), LASSBio-1735 (3) and LASSBio-2070 (10) in EGFR site.

## References

[1] Florian S., Mitchison T.J. (2016). Anti-microtubule drugs. Methods Mol. Biol..

[2] Wordeman L., Vicente J.J. (2021). Microtubule targeting agents in disease: Classic drugs, novel roles. Cancers.

[3] Stanton R.A., Gernert K.M., Nettles J.H., Aneja R. (2011). Drugs that target dynamic microtubules: A new molecular perspective. Med. Res. Rev..

[4] Krause W. (2019). Resistance to anti-tubulin agents: From vinca alkaloids to epothilones. Cancer Drug Resist..

[5] Yeung S.C., She M., Yang H., Pan J., Sun L., Chaplin D. (2007). Combination chemotherapy including combretastatin A4 phosphate and paclitaxel is effective against anaplastic thyroid cancer in a nude mouse xenograft model. J. Clin. Endocrinol. Metab..

[6] Nainwal L.M., Alam M.M., Shaquiquzzaman M., Marella A., Kamal A. (2019). Combretastatin-based compounds with therapeutic characteristics: A patent review. Expert Opin. Ther. Pat..

[7] do Amaral D.A., Cavalcanti B.C., Bezerra D.P., Ferreira P.M.P., Castro R.P., Sabino J.R., Machado C.M.L., Chammas R., Pessoa C., Sant’Anna C.M.R. (2014). Docking, synthesis and antiproliferative activity of *N*-acylhydrazone derivatives designed as combretastatin A4 analogues. PLoS ONE.

[8] Thota S., Rodrigues D.A., Pinheiro P.S.M., Lima L.M., Fraga C.A.M., Barreiro E.J. (2018). *N*-acylhydrazones as drugs. Bioorg. Med. Chem. Lett..

[9] Negi A.S., Gautam Y., Alam S., Chanda D., Luqman S., Sarkar J., Khan F., Konwar R. (2015). Natural antitubulin agents: Importance of 3,4,5-trimethoxyphenyl fragment. Bioorg. Med. Chem..

[10] Lima L.M., Alves M.A., do Amaral D.N. (2019). Homologation: A versatile molecular modification strategy to drug discovery. Curr. Top. Med. Chem..

[11] Mosman T. (1983). Rapid colorimetric assay for cellular growth and survival: Application to proliferation and cytotoxicity assays. J. Immunol. Methods.

[12] Bargou R.C., Jürchott K., Wagener C., Bergmann S., Metzner S., Bommert K., Mapara K.M.Y., Winzer K.J., Dietel M., Dörken B. (1997). Nuclear localization and increased levels of transcription factor YB-1 in primary human breast cancers are associated with intrinsic MDR1 gene expression. Nat. Med..

[13] Tsou S., Hou M., Hsu L., Chen Y., Chen Y. (2016). Gain-of-function p53 mutant with 21-bp deletion confers susceptibility to multidrug resistance in MCF-7 cells. Int. J. Mol. Med..

[14] Pokharel D., Roseblade A., Oenarto V., Lu J.F., Bebawy M. (2017). Proteins regulating the intercellular transfer and function of P-glycoprotein in multidrug-resistant cancer. Ecancermedicalscience.

[15] Borenfreund E., Puerner J.A. (1985). Toxicity determined in vitro by morphological alterations and neutral red absorption. Toxicol. Lett..

[16] Kars M.D., Işeri O.D., Gündüz U. (2011). A microarray based expression profiling of paclitaxel and vincristine resistant MCF-7 cells. Eur. J. Pharmacol..

[17] Ashraf M., Shaik T.B., Malik M.S., Syed R., Mallipeddi P.L., Vardhan M., Kamal A. (2016). Design and synthesis of cis-restricted benzimidazole and benzothiazole mimics of combretastatin A-4 as antimitotic agents with apoptosis inducing ability. Bioorg. Med. Chem. Lett..

[18] Shan Y., Zhang J., Liu Z., Wang M., Dong Y. (2011). Developments of combretastatin A-4 derivatives as anticancer agents. Curr. Med. Chem..

[19] Barreca M., Stathis A., Barraja P., Bertoni F. (2020). An overview on anti-tubulin agents for the treatment of lymphoma patients. Pharmacol. Therapeut..

[20] Gigant B., Wang C., Ravelli R.B.G., Roussi F., Steinmetz M.O., Curmi P.A., Sobel A., Knossow M. (2005). Structural basis for the regulation of tubulin by vinblastine. Nature.

[21] Steinmetz M.O., Prota A.E. (2018). Microtubule-targeting agents: Strategies to hijack the cytoskeleton. Trends Cell Biol..

[22] Sebahar P.R., Willardsen A., Anderson M.B. (2009). Anticancer agents: VTA or VDA. Curr. Bioact. Compd..

[23] Akhmanova A., Steinmetz M.O. (2015). Control of microtubule organization and dynamics: Two ends in the limelight. Nat. Rev. Mol. Cell Biol..

[24] Xiao H., Pinard P.V., Fuentes N.F., Burd B., Angeletti R., Fiser A., Horwitz S.B., Orr G.A. (2006). Insights into the mechanism of microtubule stabilization by Taxol. Proc. Natl. Acad. Sci. USA.

[25] Owens J. (2007). Determining druggability. Nat. Rev. Drug Discov..

[26] Agoni C., Olotu F.A., Ramharack P., Soliman M.E. (2020). Druggability and drug-likeness concepts in drug design: Are biomodelling and predictive tools having their say?. J. Mol. Model..

[27] Salum L.B., Mascarello A., Canevarolo R.R., Altei W.F., Laranjeira A.B., Neuenfeldt P.D., Stumpf T.R., Delatorre L.D.C., Vollmer L.L., Daghestani H.N. (2015). *N*-(1′-naphthyl)-3,4,5-trimethoxybenzohydrazide as microtubule destabilizer: Synthesis, cytotoxicity, inhibition of cell migration and *in vivo* activity against acute lymphoblastic leukemia. Eur. J. Med. Chem..

[28] Wu Y.L., Tsuboi M., He J., John T., Grohe C., Majem M., Goldman J.W., Laktionov K., Kim S.W., Kato T. (2020). Osimertinib in resected EGFR-mutated non–small-cell lung cancer. N. Engl. J. Med..

[29] McLoughlin E.C., O´Boyle N.M. (2020). Colchicine-binding site inhibitors from chemistry to clinic: A review. Pharmaceuticals.

[30] Li W., Sun H., Xu S., Zhu Z., Xu J. (2017). Tubulin inhibitors targeting the colchicine binding site: A perspective of privileged structures. Future Med. Chem..

[31] Yang J., Yan W., Yu Y., Wang Y., Yang T., Xue L., Yuan X., Long C., Liu Z., Chen X. (2018). The compound millepachine and its derivatives inhibit tubulin polymerization by irreversibly binding to the colchicine-binding site in β-tubulin. J. Biol. Chem..

[32] Wang J., Miller D.D., Li W. (2022). Molecular interactions at the colchicine binding site in tubulin: An X-ray crystallography perspective. Drug Dis. Today.

[33] Chen H., Deng S., Albadari N., Yun M.K., Zhang S., Li Y., Ma D., Parke D.N., Yang L., Seagroves T.N. (2021). Design, synthesis, and biological evaluation of stable colchicine-binding site tubulin inhibitors 6-aryl-2-benzoyl-pyridines as potential anticancer agents. J. Med. Chem..

[34] Debs G.E., Cha M., Liu X., Sindelar C.V. (2020). Dynamic and asymmetric fluctuations in the microtubule wall captured by high-resolution cryoelectron microscopy. Proc. Natl. Acad. Sci. USA.

[35] Pawar D.M., Khali A.A., Hooks D.R., Collins K., Elliott T., Stafford J., Smith L., Noe E.A. (1998). E and Z conformations of esters, thiol esters, and amides. J. Am. Chem. Soc..

[36] Field J.J., Díaz J.F., Miller J.H. (2013). The binding sites of microtubule-stabilizing agents. Chem. Biol..

[37] Mitra A., Sept D. (2008). Taxol allosterically alters the dynamics of the tubulin dimer and increases the flexibility of microtubules. Biophys. J..

[38] Korb O., Stützle T., Exner T.E. (2009). Empirical scoring functions for advanced protein−ligand docking with PLANTS. J. Chem. Inf. Model..

[39] Kümmerle A.E., Raimundo J.M., Leal C.M., da Silva G.S., Balliano T.L., Pereira M.A., de Simone C.A., Sudo R.T., Zapata-Sudo G., Fraga C.A.M. (2009). Studies towards the identification of putative bioactive conformation of potent vasodilator arylidene *N*-acylhydrazone derivatives. Eur. J. Med. Chem..

[40] Barreiro E.J., Kümmerle A.E., Fraga C.A.M. (2011). The methylation effect in medicinal chemistry. Chem. Rev..

[41] Heppner D.E., Günther M., Wittlinger F., Laufer S.A., Eck M.J. (2020). Structural basis for EGFR mutant inhibition by trisubstituted imidazole inhibitors. J. Med. Chem..

[42] He J., Zhou Z., Sun X., Yang Z., Zheng P., Xu S., Zhu W. (2021). The new opportunities in medicinal chemistry of fourth-generation EGFR inhibitors to overcome C797S mutation. Eur. J. Med. Chem..

[43] Patel H., Pawara R., Ansari A., Surana S. (2017). Recent updates on third generation EGFR inhibitors and emergence of fourth generation EGFR inhibitors to combat C797S resistance. Eur. J. Med. Chem..

[44] D’Alessandro R., Refolo M.G., Lippolis C., Carella N., Messa C., Cavallini A., Carr B.I. (2015). Modulation of Regorafenib effects on HCC cell lines by epidermal growth fator. Cancer Chemother. Pharmacol..

[45] Moskot M., Gabig-Cimińska M., Jakóbkiewicz-Banecka J., Węsierska M., Bocheńska K., Węgrzyn G. (2016). Cell cycle is disturbed in mucopolysaccharidosis type II fibroblasts, and can be improved by genistein. Gene.

[46] Filgueiras M.C., Morrot A., Soares P.M.G., Costa M.L., Mermelstein C. (2013). Effects of 5-fluorouracil in nuclear and cellular morphology, proliferation, cell cycle, apoptosis, cytoskeletal and caveolar distribution in primary cultures of smooth muscle cells. PLoS ONE.

[47] Stamos J., Sliwkowski M.X., Eigenbrot C. (2002). Structure of the epidermal growth factor receptor kinase domain alone and in complex with a 4-anilinoquinazoline inhibitor. J. Biol. Chem..

[48] Yan X.E., Ayaz P., Zhu S.J., Zhao P., Liang L., Zhang C.H., Wu Y.C., Li J.L., Choi H.G., Huang X. (2020). Structural basis of AZD9291 selectivity for EGFR T790M. J. Med. Chem..

