# Supplementary material for: Discovery of Putative Dual Inhibitor of Tubulin and EGFR by Phenotypic Approach on LASSBio-1586 Homologs
Source: Pharmaceuticals (Basel). 2022 Jul 23;15(8):913. doi: 10.3390/ph15080913 (PMC9394307; doi:10.3390/ph15080913)
Supplement: Supplementary file 1 [file pharmaceuticals-15-00913-s001.zip › pharmaceuticals-1817477-supplementary.pdf]

Supplementary Material

DISCOVERY OF PUTATIVE DUAL INHIBITORS OF TUBULIN AND EGFR BY PHENOTYPIC APPROACH ON LASSBio-1586 HOMOLOGS.

Gisele Barbosa[1,3], Luis Gabriel Valdivieso Gelves[1,3], Caroline Marques Xavier Costa[1,3], Lucas Silva Franco[1,3], João Alberto Lins de Lima[1,3], Cristiane Aparecida e Silva[1,3], John Douglas Teixeira[2], Claudia dos Santos Mermelstein[2], Eliezer J. Barreiro[1,3], Lidia Moreira Lima[1,3].

[1] Instituto Nacional de Ciência e Tecnologia de Fármacos e Medicamentos (INCT-INOFAR), Universidade Federal Do Rio de Janeiro, Laboratório de Avaliação e Síntese de Substâncias Bioativas (LASSBio®), CCS, Cidade Universitária, P.O. Box 68024, 21941-971, Rio de Janeiro, RJ, Brasil

[2] Laboratório de Diferenciação Muscular e Citoesqueleto, Instituto de Ciências Biomédicas, Universidade Federal do Rio de Janeiro, Rio de Janeiro, RJ, Brasil

[3] Programa de Pós-graduação em Farmacologia e Química Medicinal, Instituto de Ciências Biomédicas, Universidade Federal do Rio de Janeiro, Rio de Janeiro, RJ, Brasil

Table of Contents

NMR and IR Spectrum data.. 2
Selectivity Index (SI) data 26
Molecular Docking Studies..27

NMR and IR Spectrum data

All compounds were characterized by NMR and IR techniques and the spectra of the synthesized compounds are shown below:

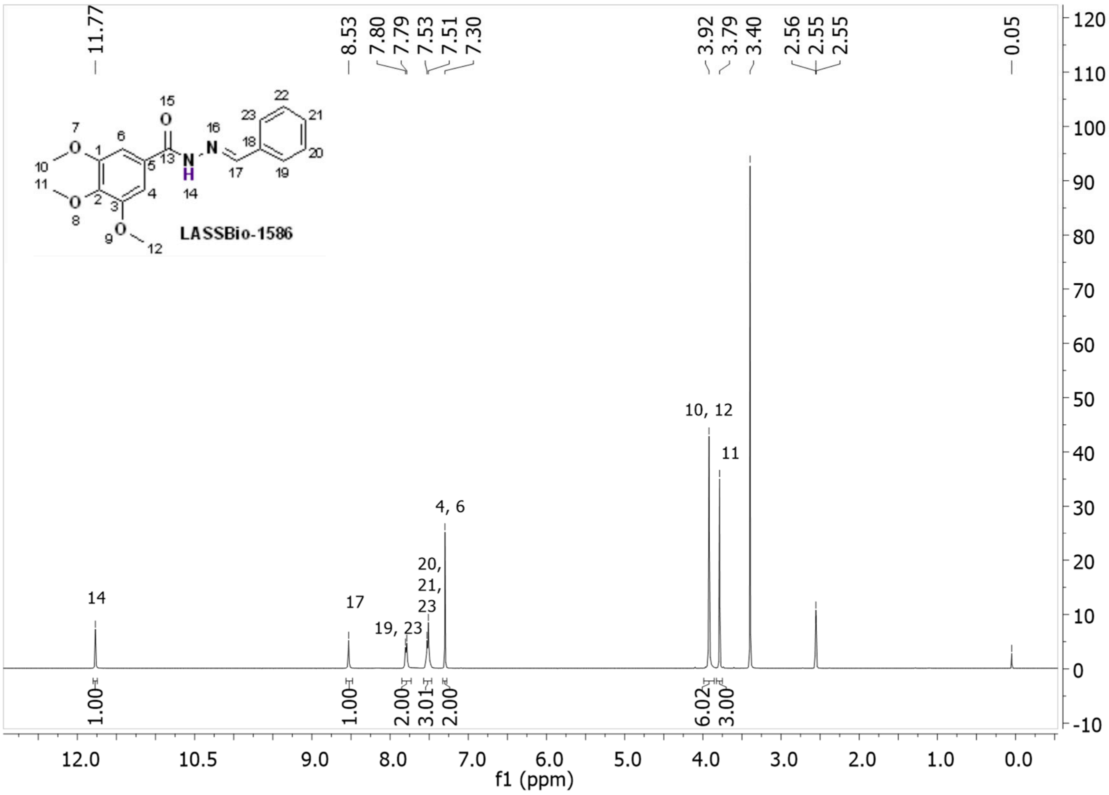

Figure S1. ^{1}H NMR spectra of (E)-N'benzylidene-3,4,5-trimetoxybenzohydrazide (**2**) (500 MHz, DMSO-d_6).

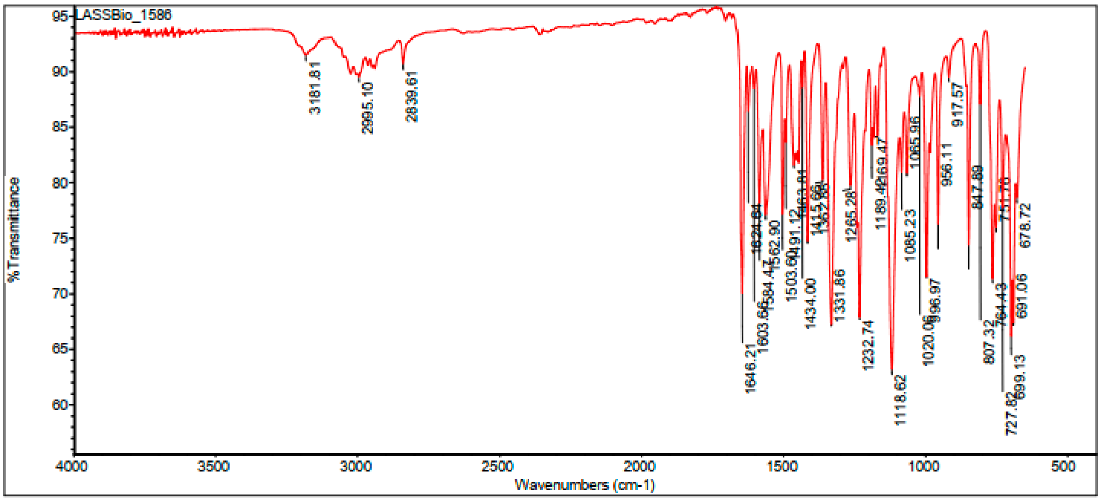

Figure S2. Absorption spectra in the infrared region of *(E)-N'*benzylidene-3,4,5-trimetoxybenzohydrazide (**2**) (ATR).

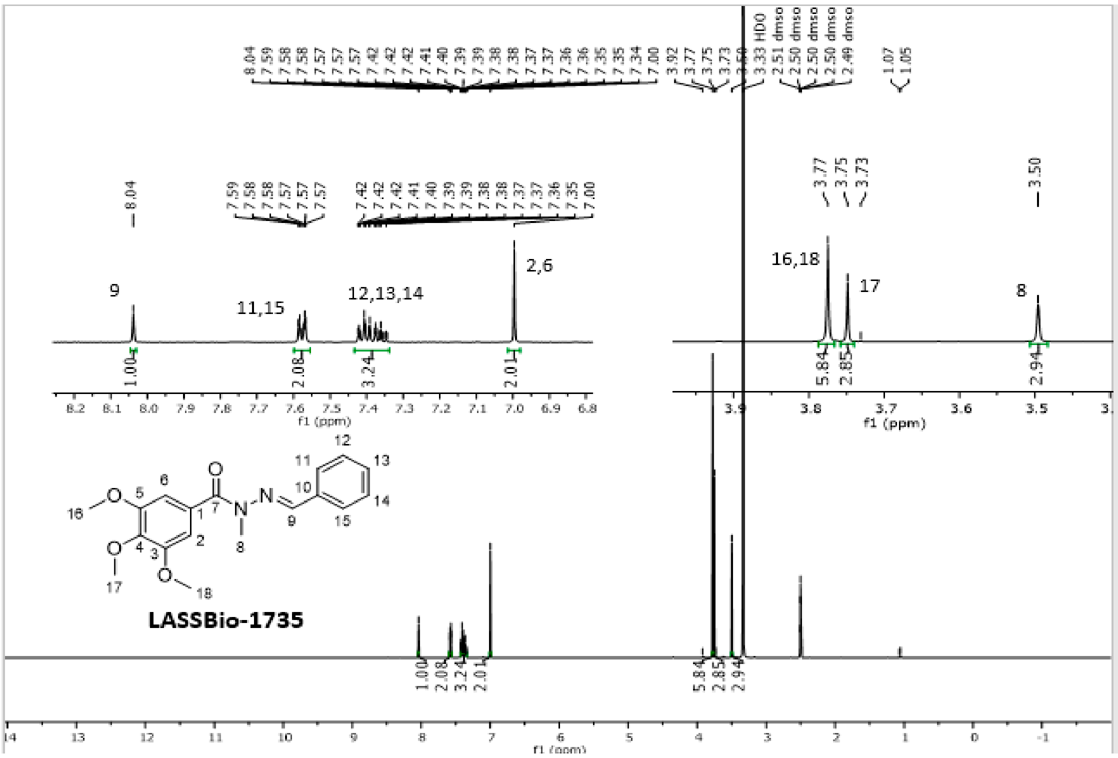

Figure S3. ^{1}H NMR spectra of *(E)-N′*-benzylidene-3,4,5-trimethoxy-*N*-methylbenzohydrazide (**3**) (500 MHz, DMSO-d_6).

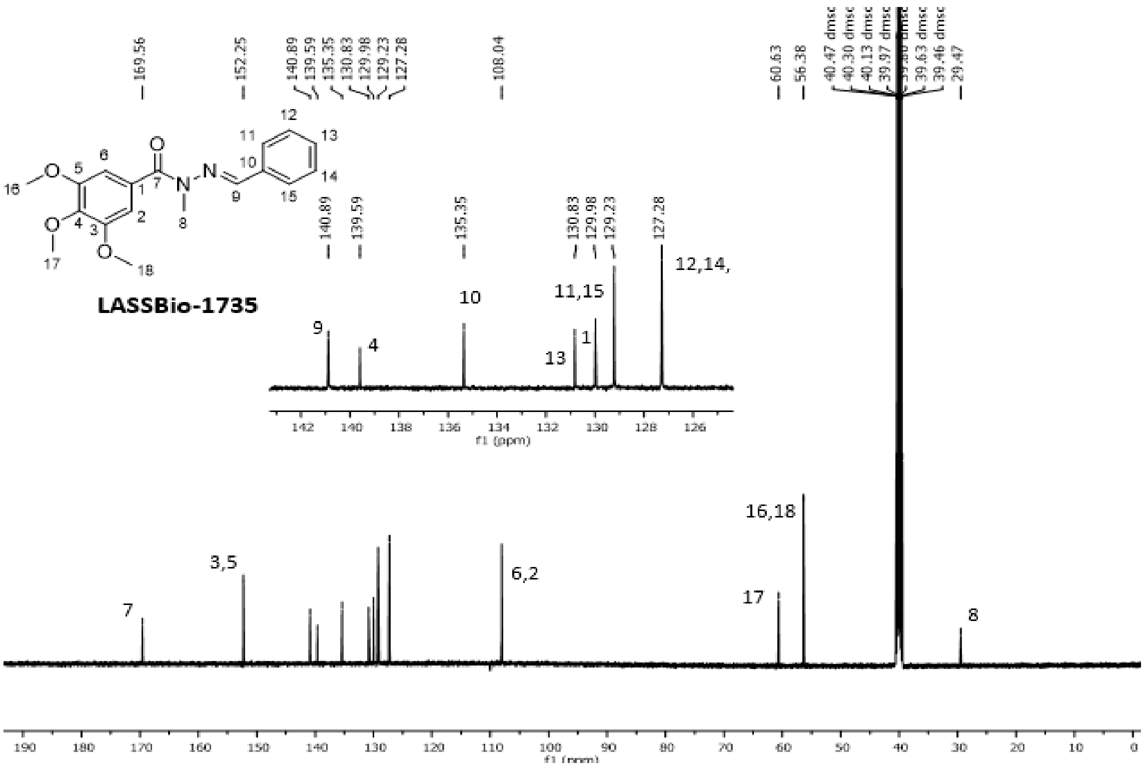

Figure S4. ^{13}C NMR spectra of *(E)-N′*-benzylidene-3,4,5-trimethoxy-*N*-methylbenzohydrazide (**3**) (125 MHz, DMSO-d_6).

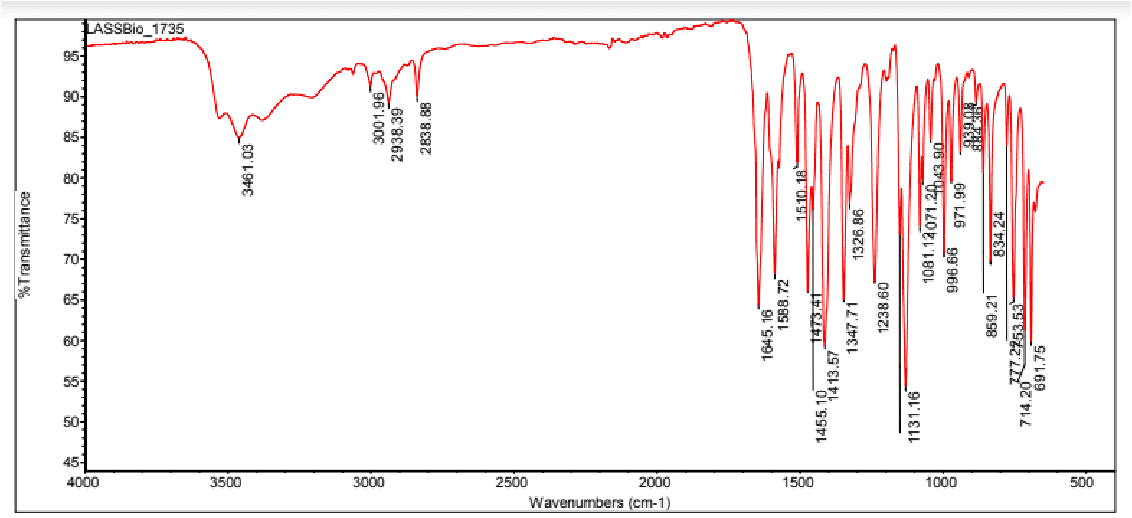

Figure S5. Absorption spectra in the infrared region of *(E)*-*N'*-benzylidene-3,4,5-trimethoxy-*N*-methylbenzohydrazide (**3**) (ATR).

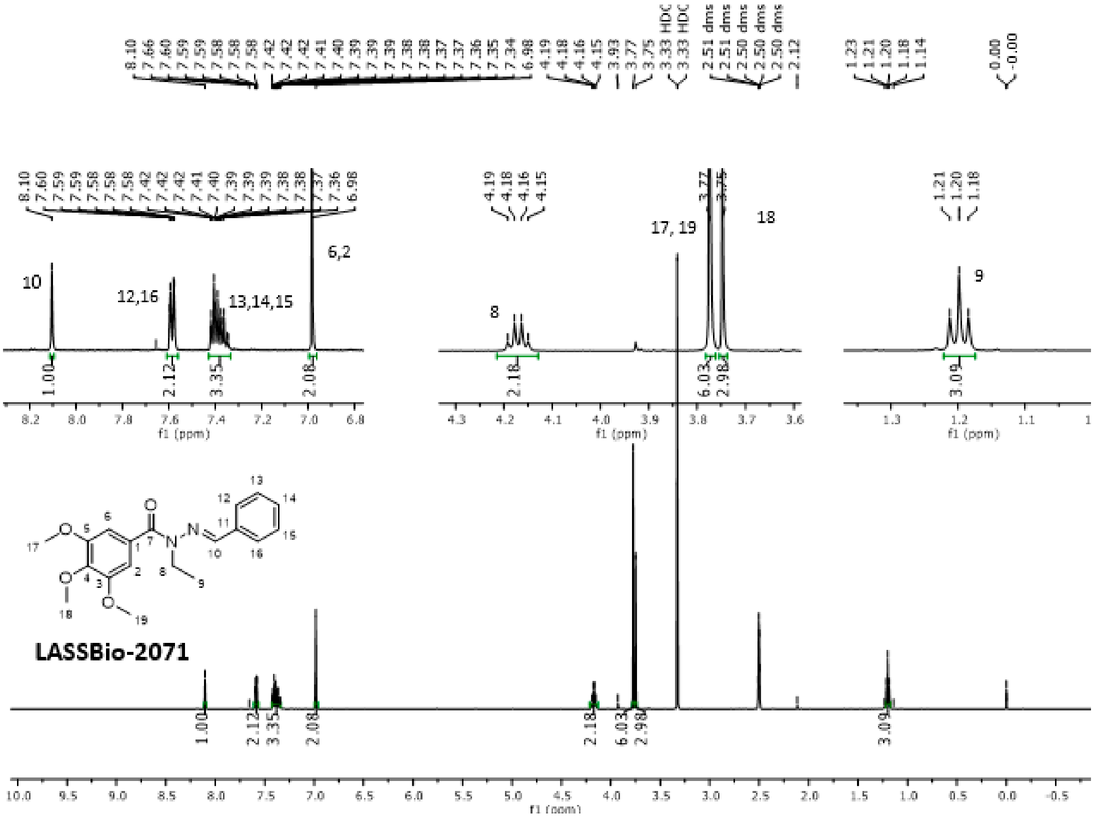

Figure S6. ^{1}H NMR spectra of *(E)*-*N'*-benzylidene-*N*-ethyl-3,4,5-trimethoxybenzohydrazide (**4**) (500 MHz, DMSO-d_6).

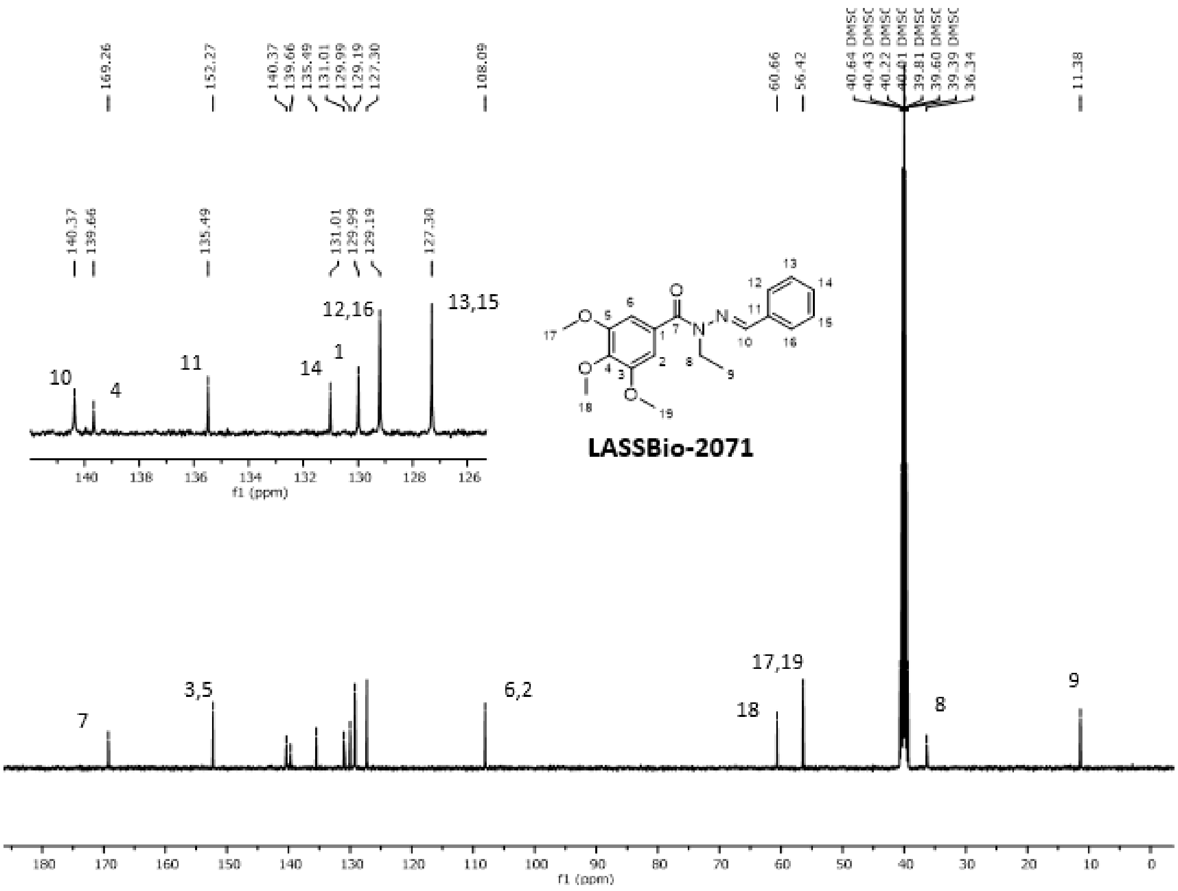

Figure S7. ^{13}C NMR spectra of *(E)*-*N'*-benzylidene-*N*-ethyl-3,4,5-trimethoxybenzohydrazide (**4**) (125 MHz, DMSO-*d*$_6$).

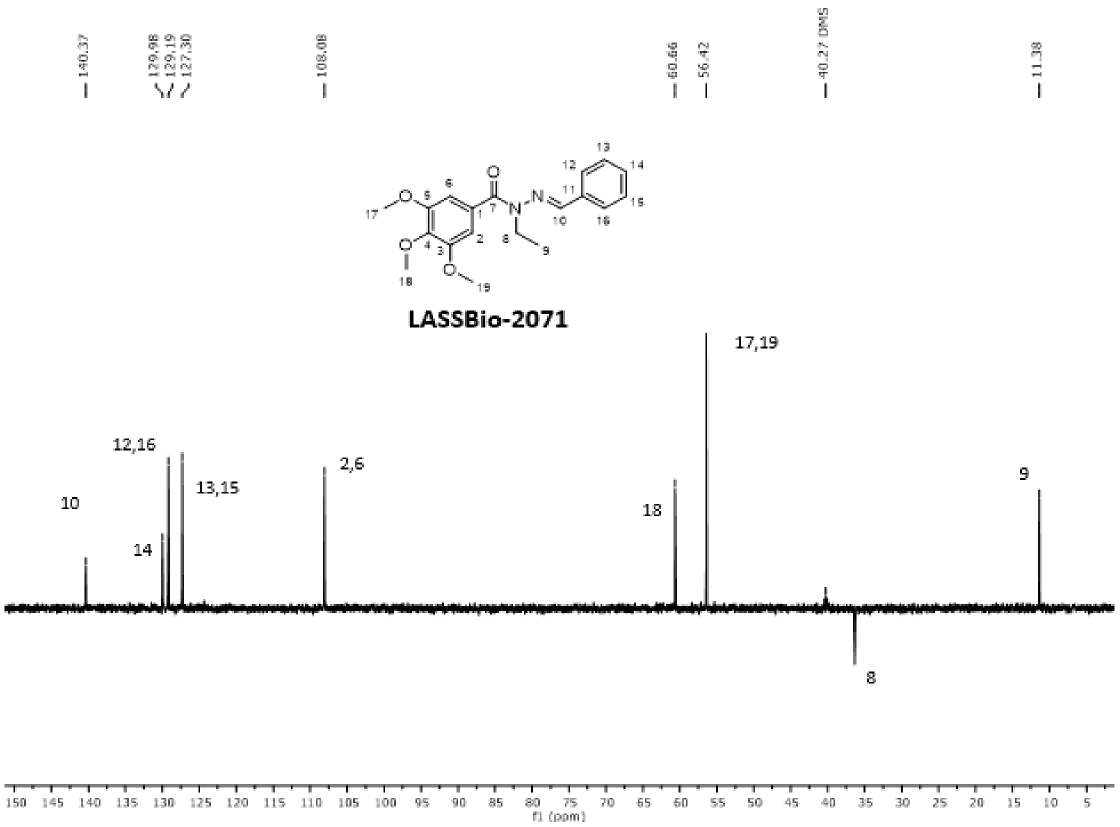

Figure S8. ^{13}C DEPT-135 NMR spectra of *(E)-N'*-benzylidene-*N*-ethyl-3,4,5-trimethoxybenzohydrazide (**4**) (126 MHz, DMSO-d_6).

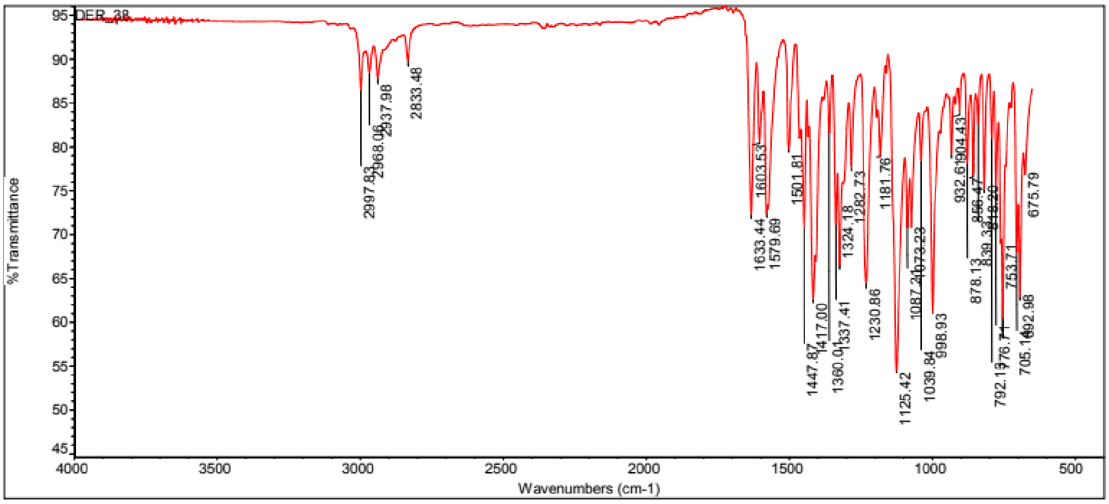

Figure S9. Absorption spectra in the infrared region of *(E)-N'*-benzylidene-*N*-ethyl-3,4,5-trimethoxybenzohydrazide (**4**) (ATR).

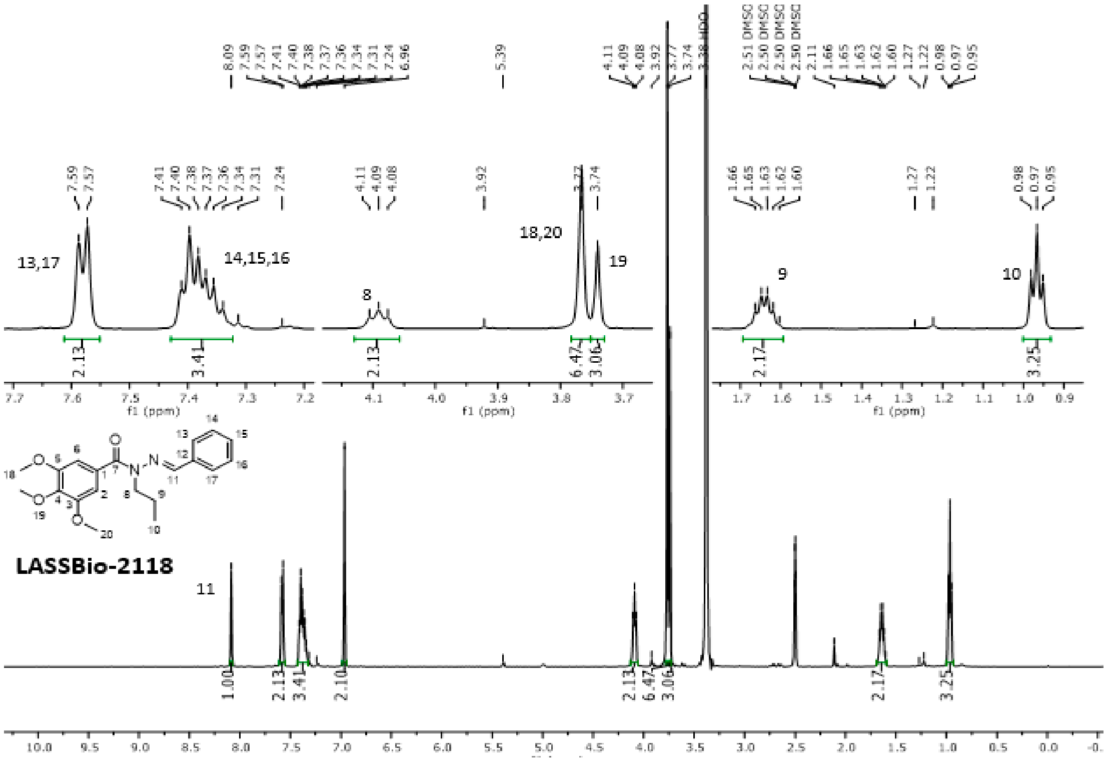

Figure S10. ^{1}H NMR spectra of *(E)-N'*-benzylidene-3,4,5-trimethoxy-*N*-propylbenzohydrazide (**5**) (500 MHz, DMSO-d$_6$).

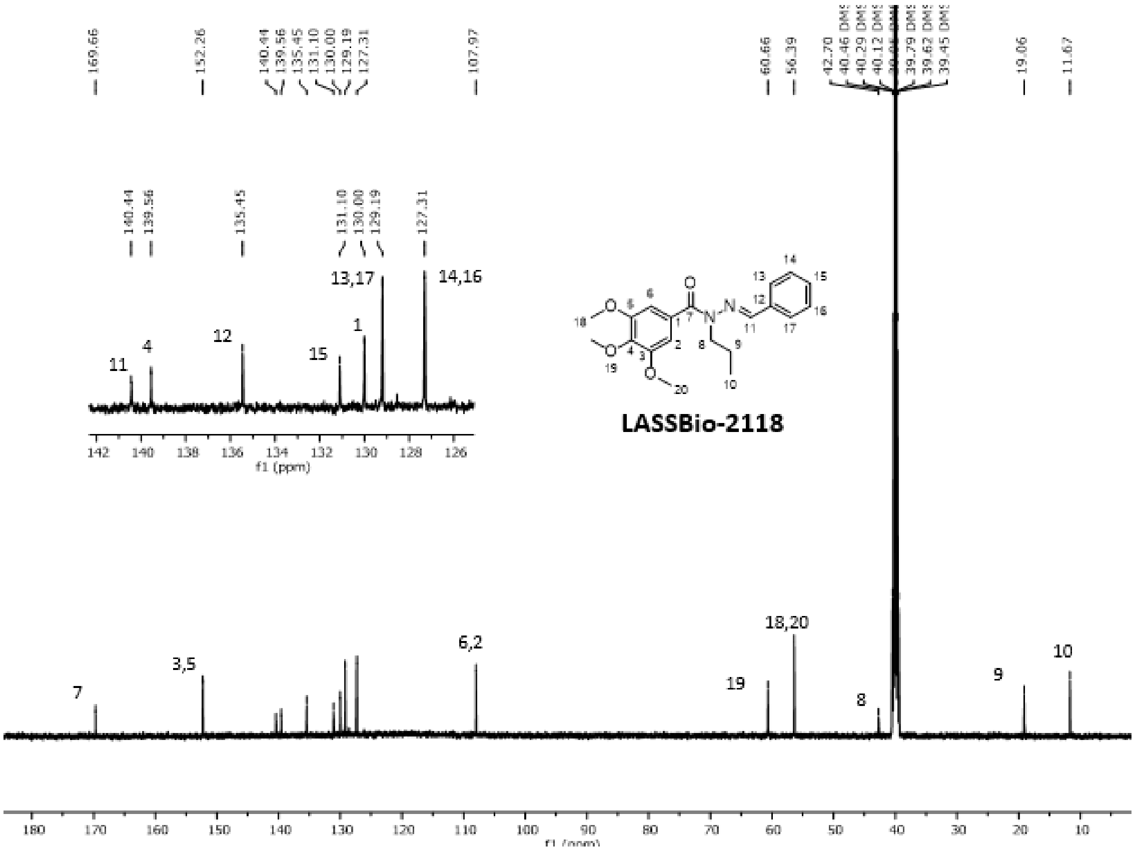

Figure S11. ^{13}C NMR spectra of *(E)*-*N'*-benzylidene-3,4,5-trimethoxy-*N*-propylbenzohydrazide (**5**) (125 MHz, DMSO-*d*$_6$,).

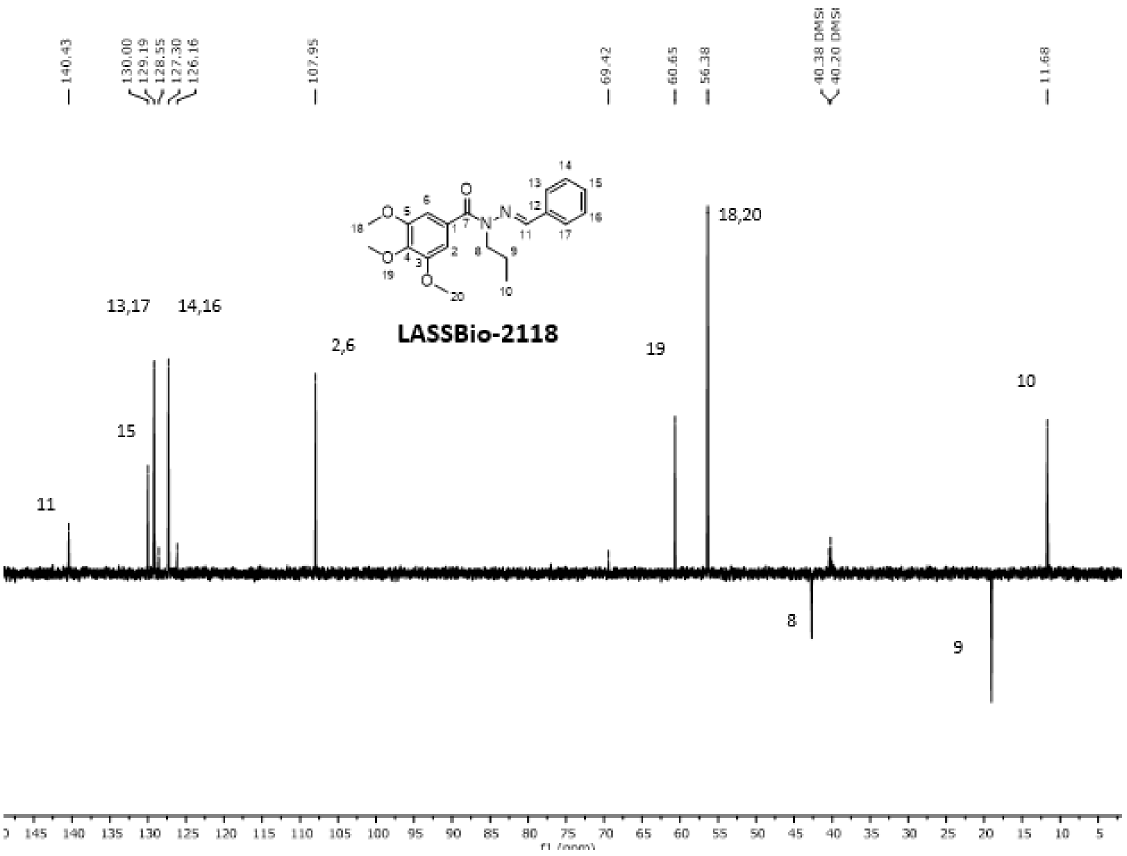

Figure S12. ^{13}C DEPT-135 NMR spectra of *(E)-N'*-benzylidene-3,4,5-trimethoxy-*N*-propylbenzohydrazide (**5**) (126 MHz, DMSO-d_6,).

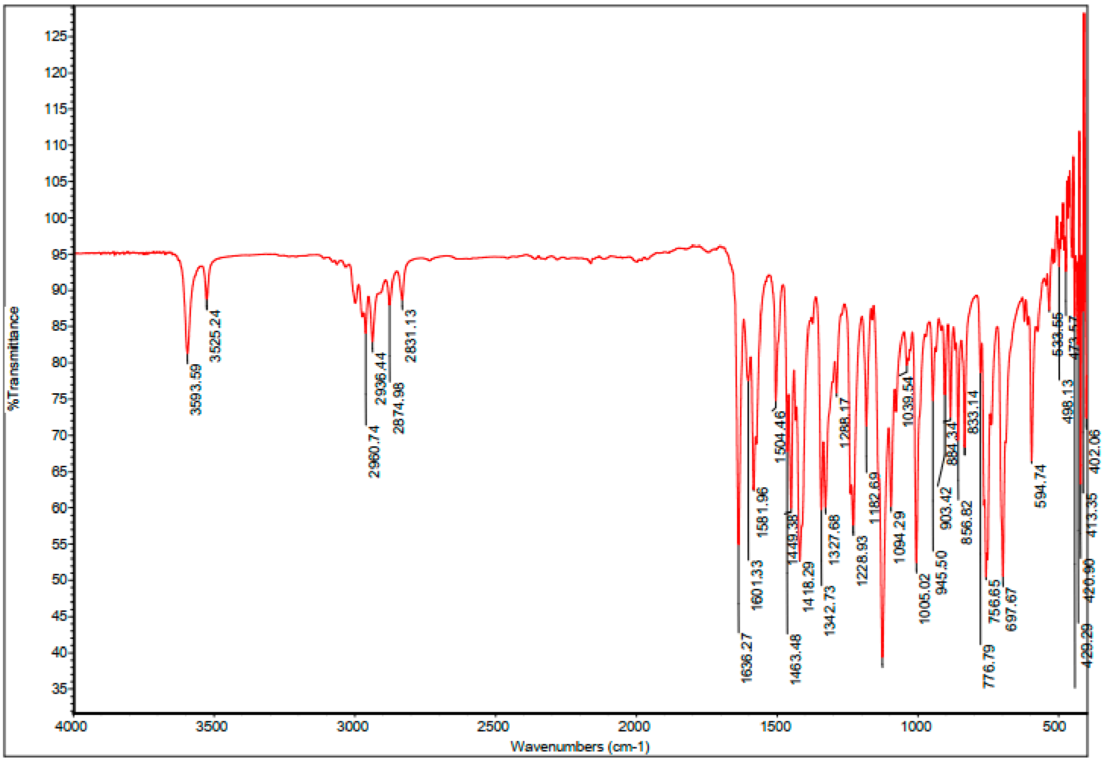

Figure S13. Absorption spectra in the infrared region of *(E)-N'*-benzylidene-3,4,5-trimethoxy-*N*-propylbenzohydrazide (**5**) (ATR).

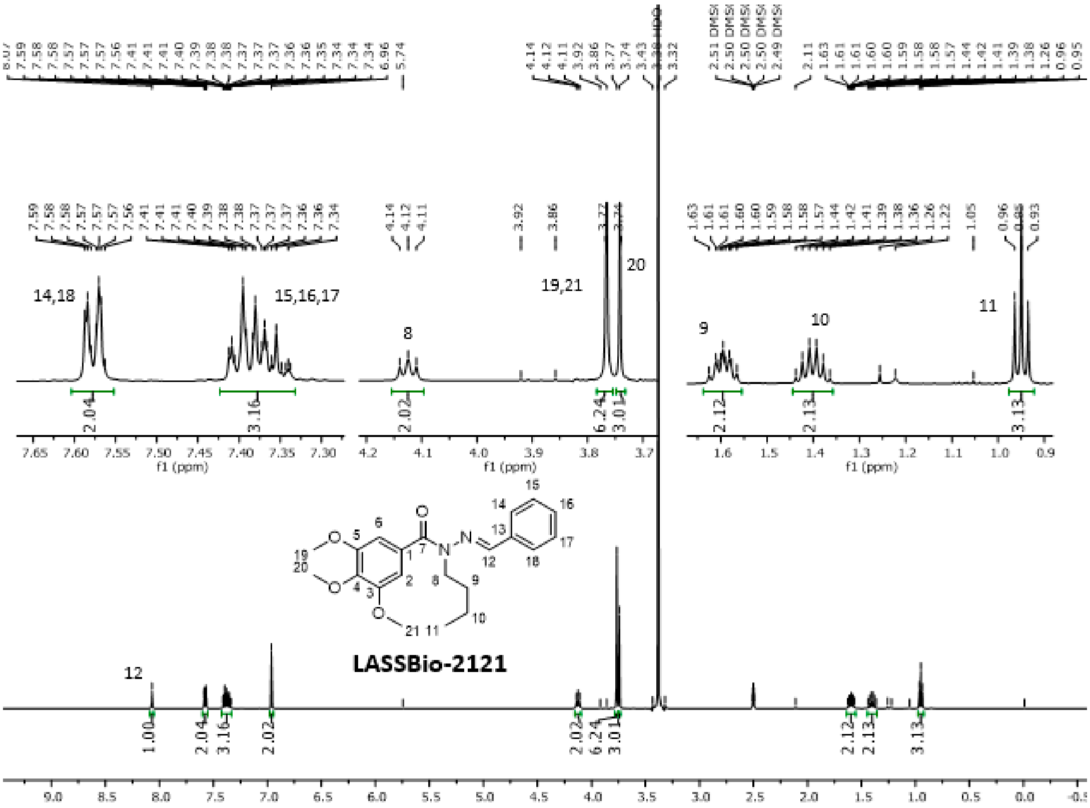

Figure S14. ^{1}H NMR spectra of *(E)-N'*-benzylidene-*N*-butyl-3,4,5-trimethoxybenzohydrazide (**6**) (500 MHz, DMSO-d_6).

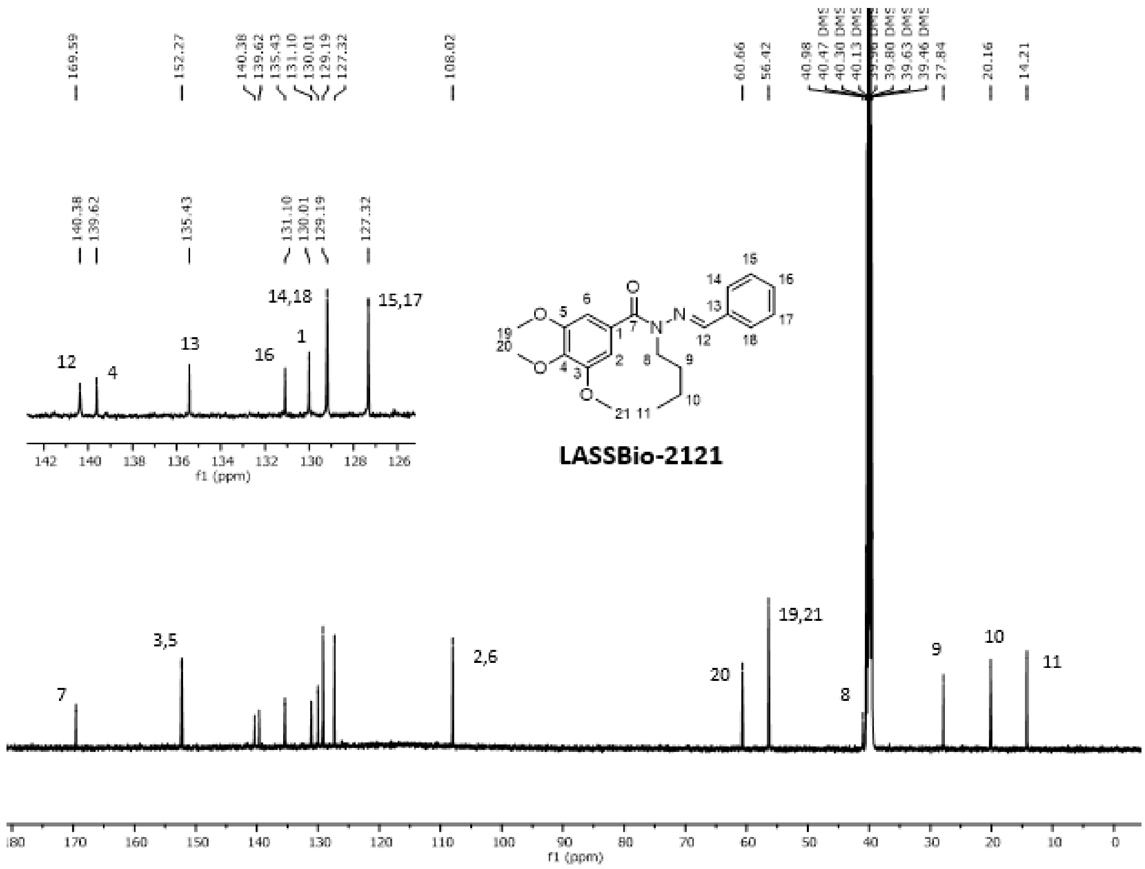

Figure S15. ^{13}C NMR specta of *(E)-N'*-benzylidene-*N*-butyl-3,4,5-trimethoxybenzohydrazide (**6**) (125 MHz, DMSO-*d*$_6$).

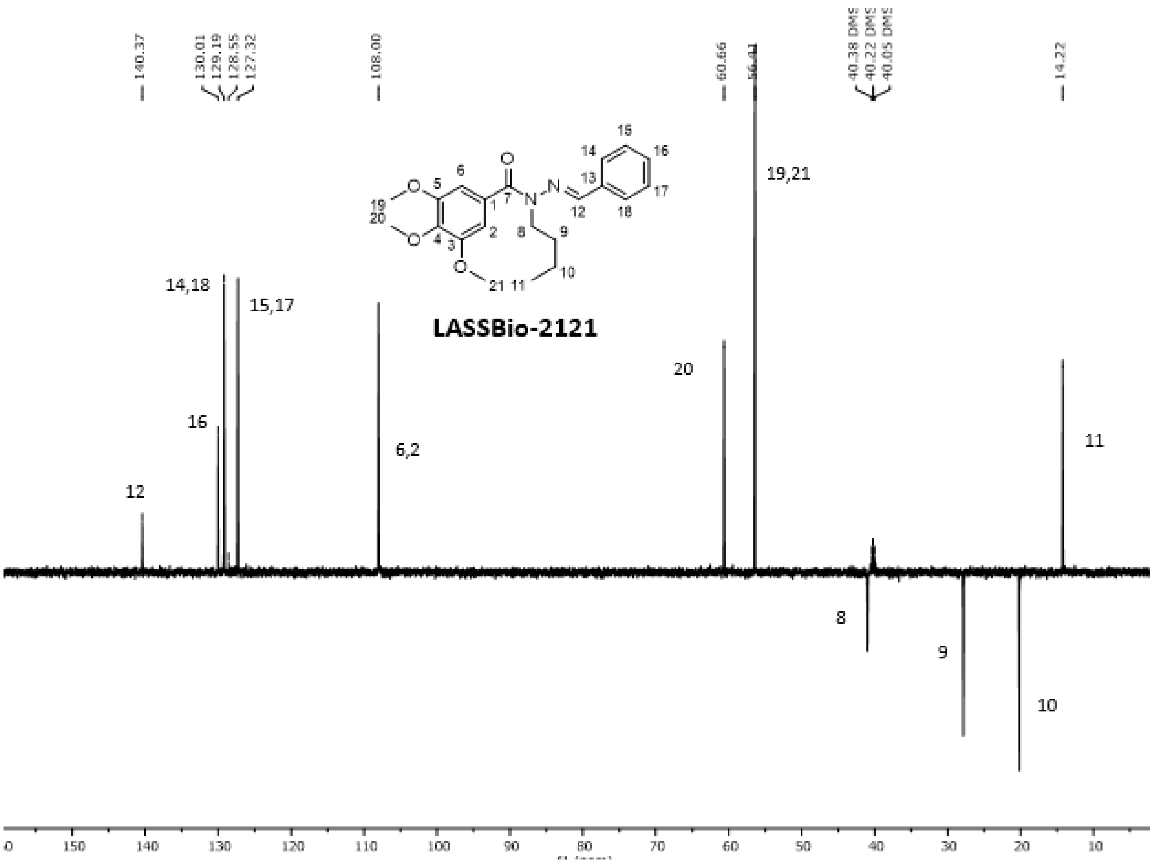

Figure S16. ^{13}C DEPT-135 NMR spectra of *(E)*-*N'*-benzylidene-*N*-butyl-3,4,5-trimethoxybenzohydrazide (**6**) (126 MHz, DMSO-d_6).

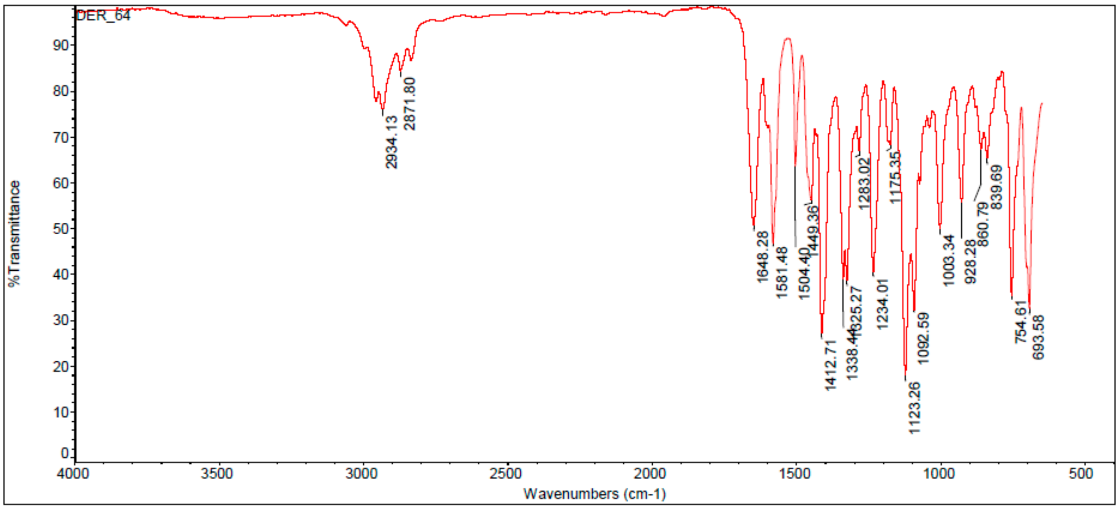

Figure S17. Absorption spectra in the infrared region of *(E)*-*N'*-benzylidene-*N*-butyl-3,4,5-trimethoxybenzohydrazide (**6**) (ATR).

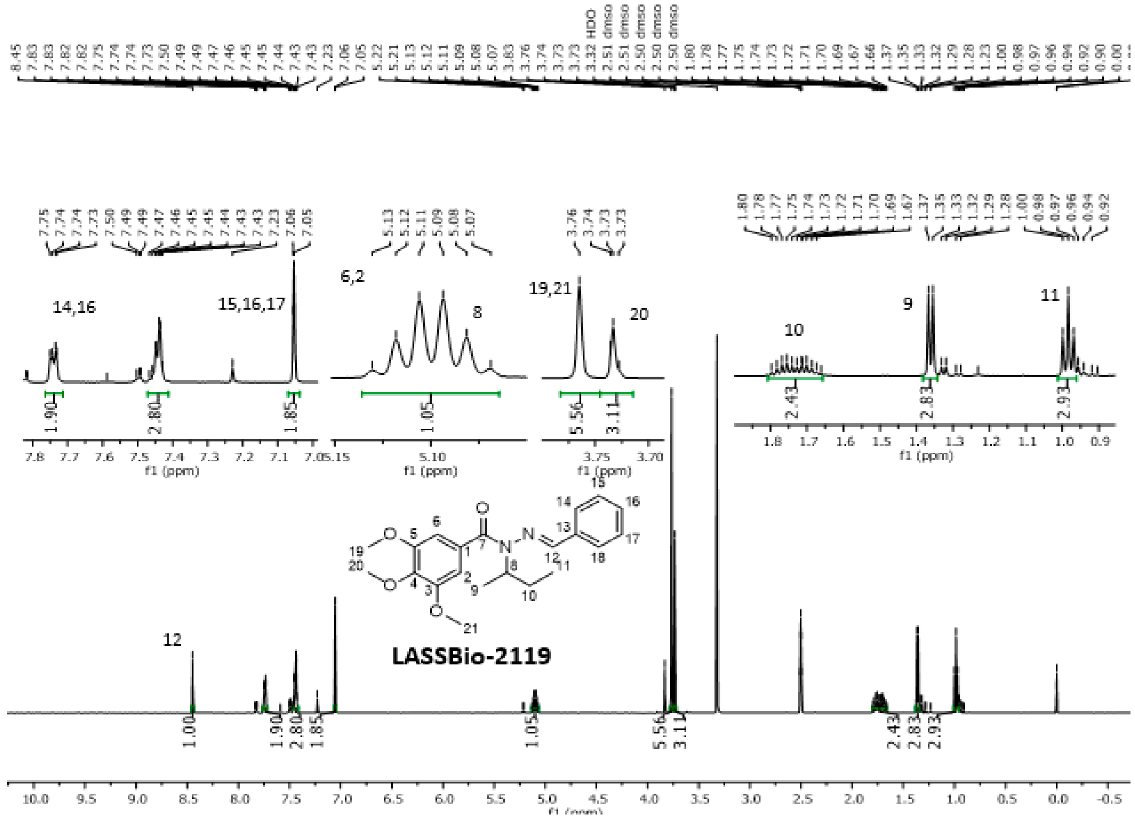

Figure S18. ^{1}H NMR spectra of *(E)-N'*-benzylidene-*N*-(sec-butyl)-3,4,5-trimethoxybenzohydrazide (**7**) (500 MHz, DMSO-d_6).

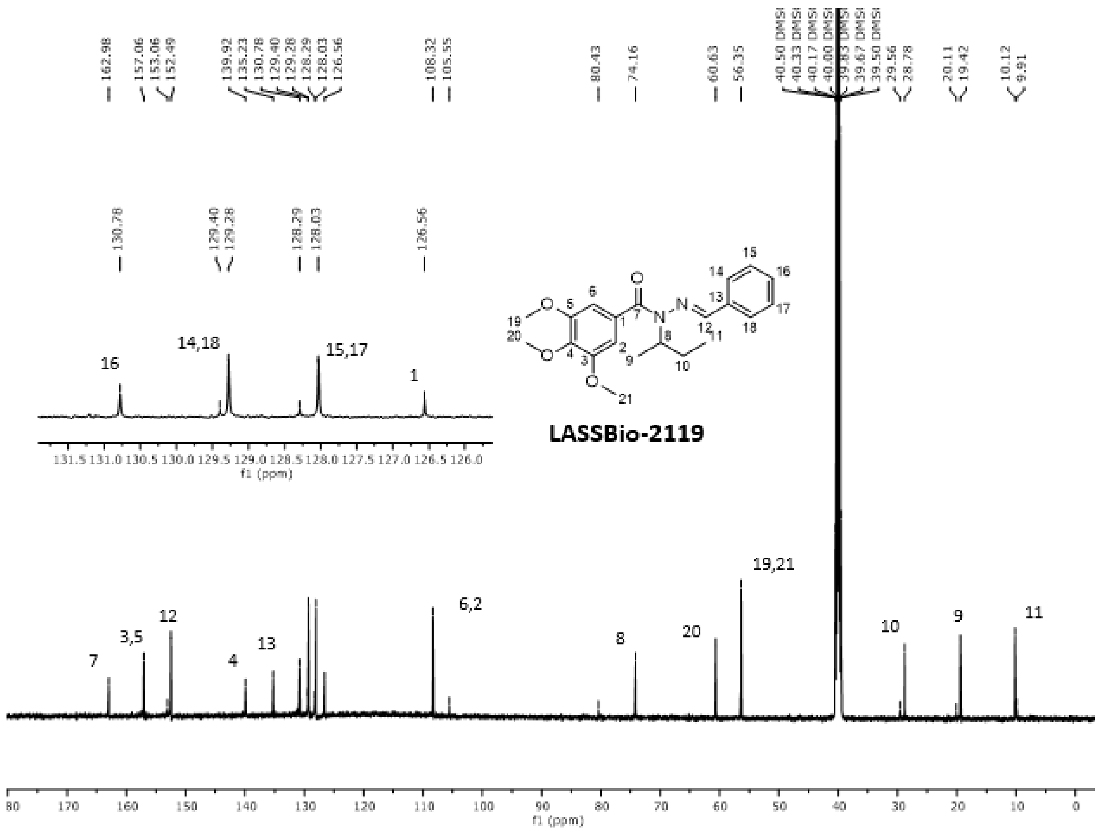

Figure S19. ^{13}C NMR spectraof *(E)-N'*-benzylidene-*N*-(sec-butyl)-3,4,5-trimethoxybenzohydrazide (**7**) (125 MHz, DMSO-*d$_6$*).

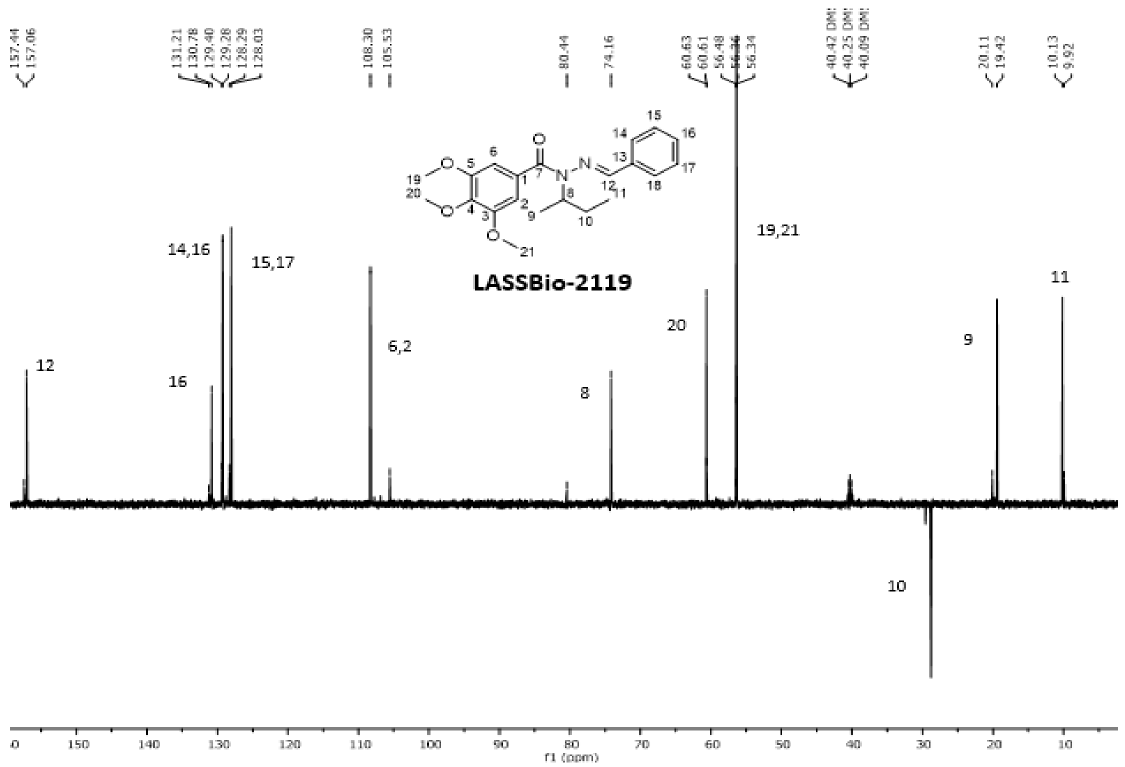

Figure S20. ^{13}C DEPT-135 NMR spectra of *(E)-N'*-benzylidene-*N*-(sec-butyl)-3,4,5-trimethoxybenzohydrazide (**7**) (126 MHz, DMSO-*d$_6$*).

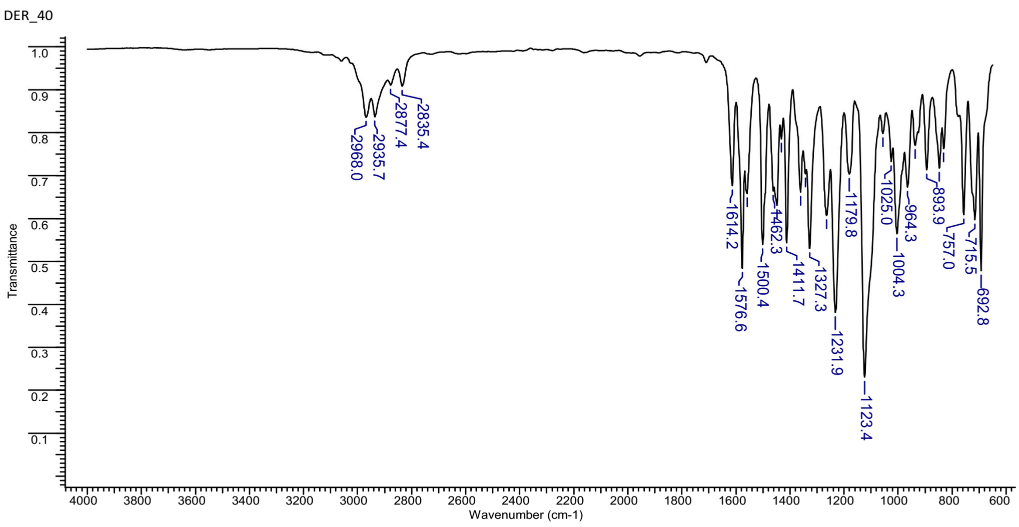

Figure S21. Absorption spectra in the infrared region of *(E)-N'*-benzylidene-*N*-(sec-butyl)-3,4,5-trimethoxybenzohydrazide (**7**) (ATR).

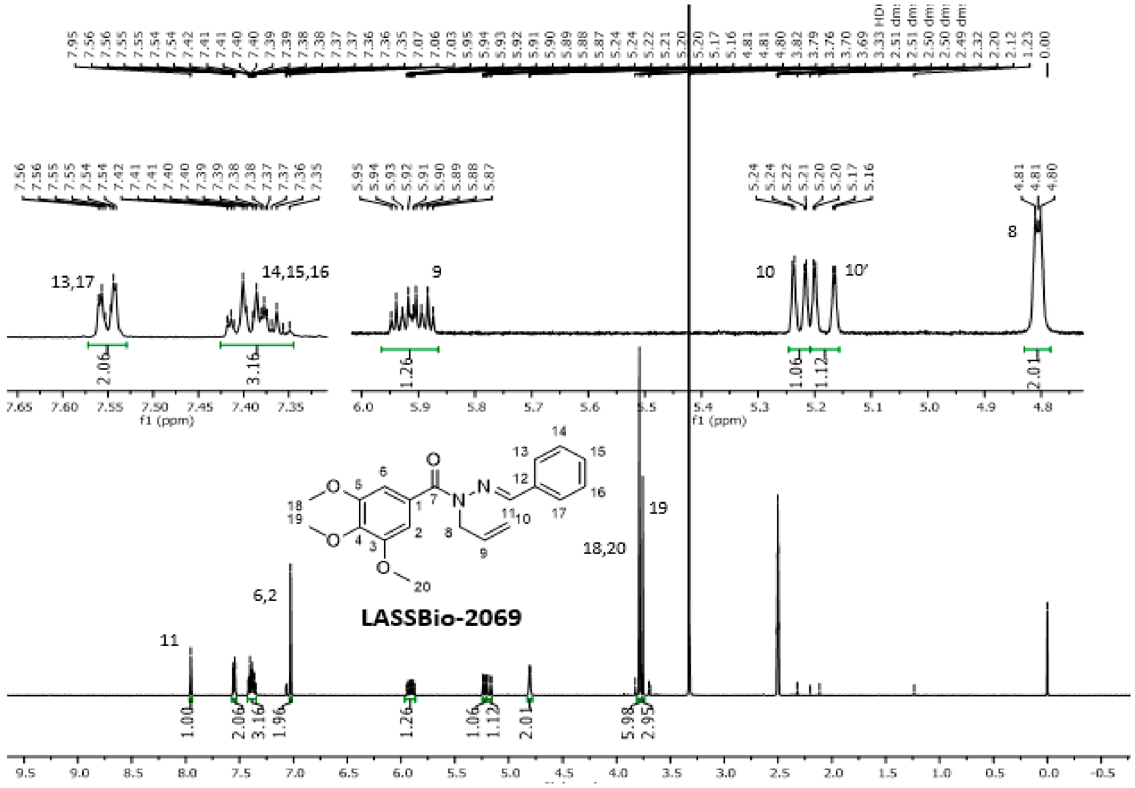

Figure S22. ^{1}H NMR spectra of *(E)-N*-allyl-*N'*-benzylidene-3,4,5-trimethoxybenzohydrazide (**8**) (400 MHz, DMSO-d_6).

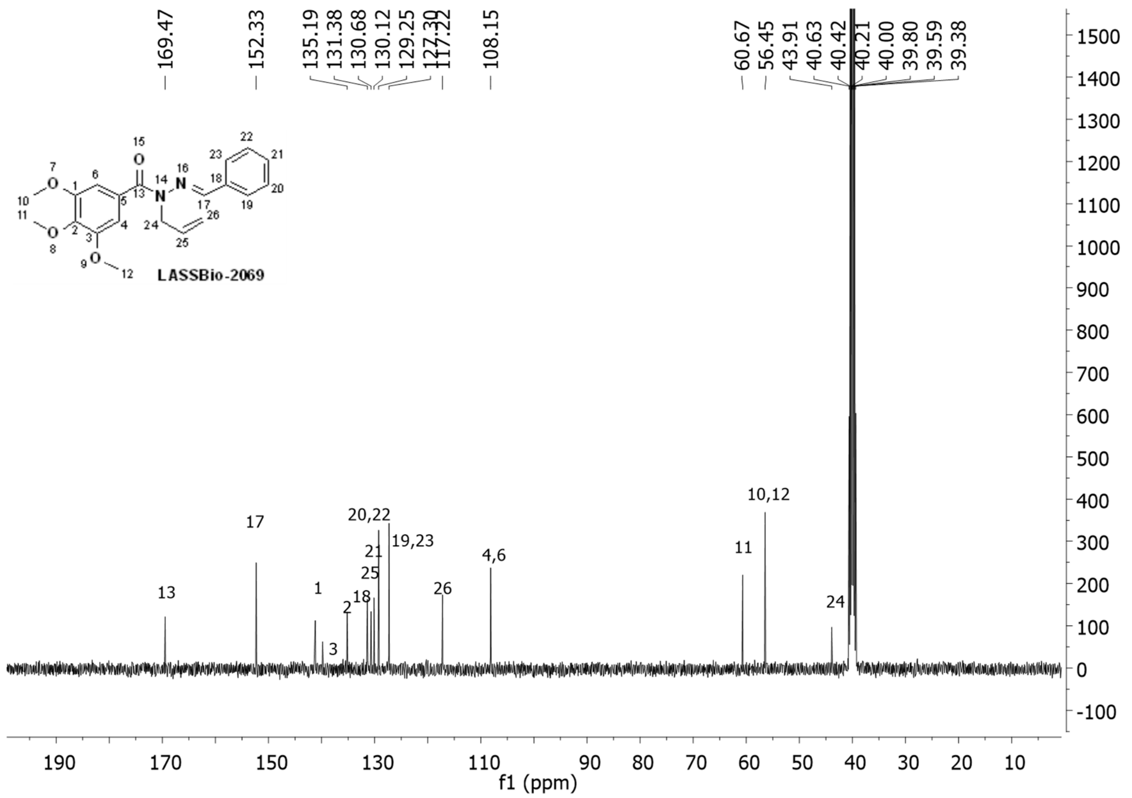

Figure S23. ^{13}C NMR spectra of *(E)*-*N*-allyl-*N'*-benzylidene-3,4,5-trimethoxybenzohydrazide (**8**) (100 MHz, DMSO-d_6).

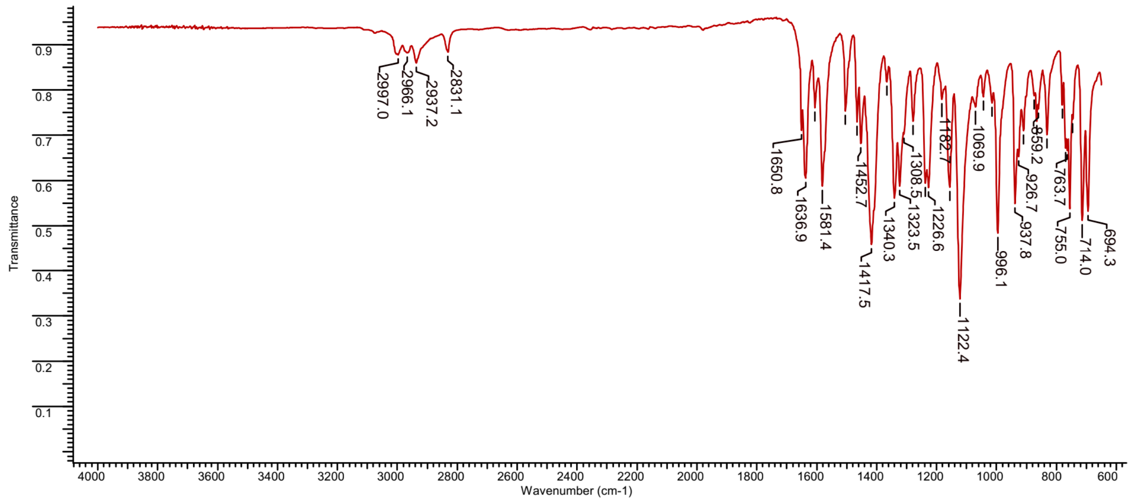

Figure S24. Absorption spectra in the infrared region of *(E)*-*N*-allyl-*N'*-benzylidene-3,4,5-trimethoxybenzohydrazide (**8**) (ATR).

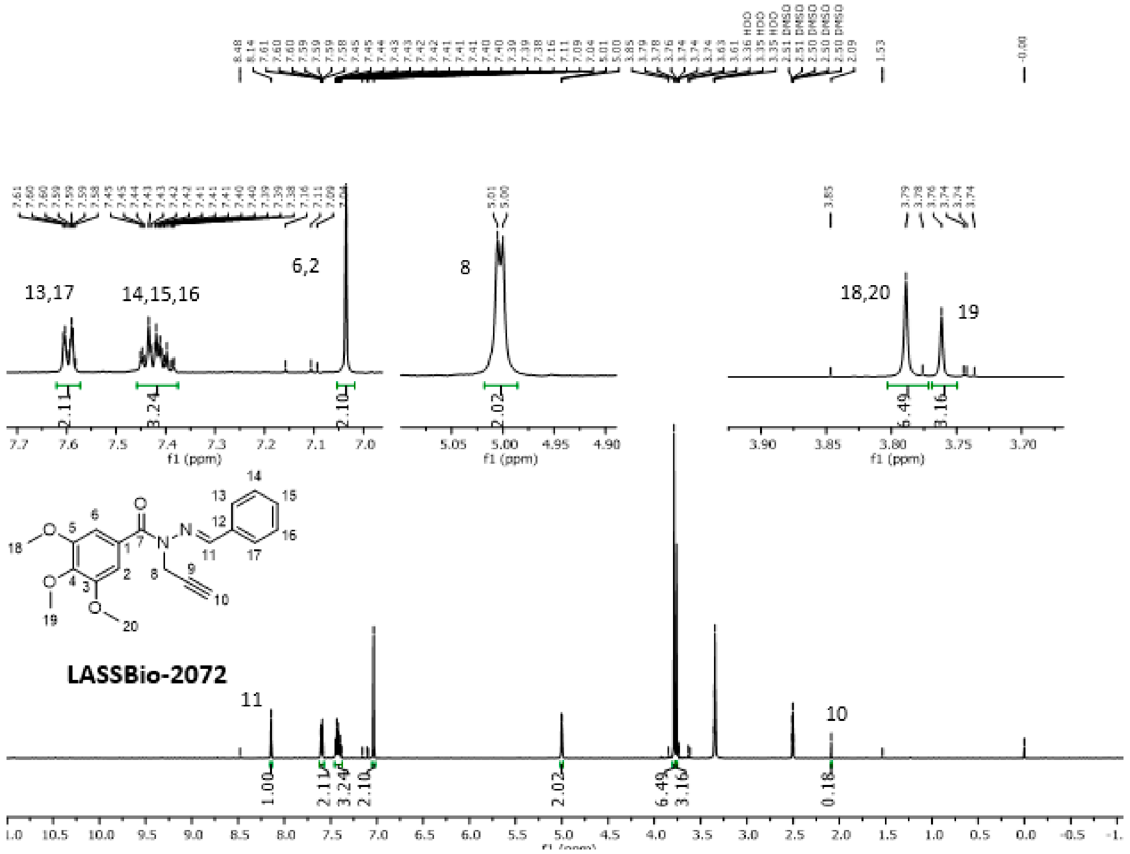

Figure S25. ^{1}H NMR spectra of *(E)-N'*-benzylidene-3,4,5-trimethoxy-*N*-(prop-2-yn-1-yl)benzohydrazide (**9**) (500 MHz, DMSO-d_6).

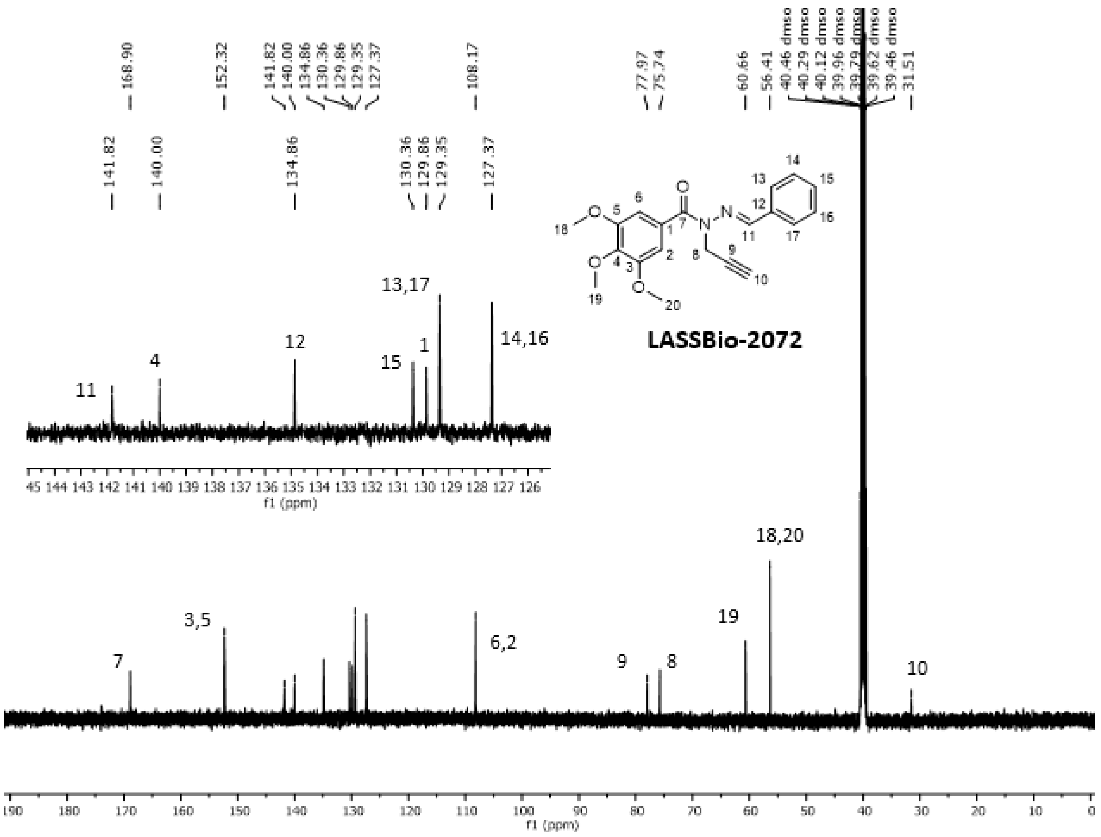

Figure S26. ^{13}C NMR spectra of *(E)-N'*-benzylidene-3,4,5-trimethoxy-*N*-(prop-2-yn-1-yl)benzohydrazide (**9**) (125 MHz, DMSO-d_6).

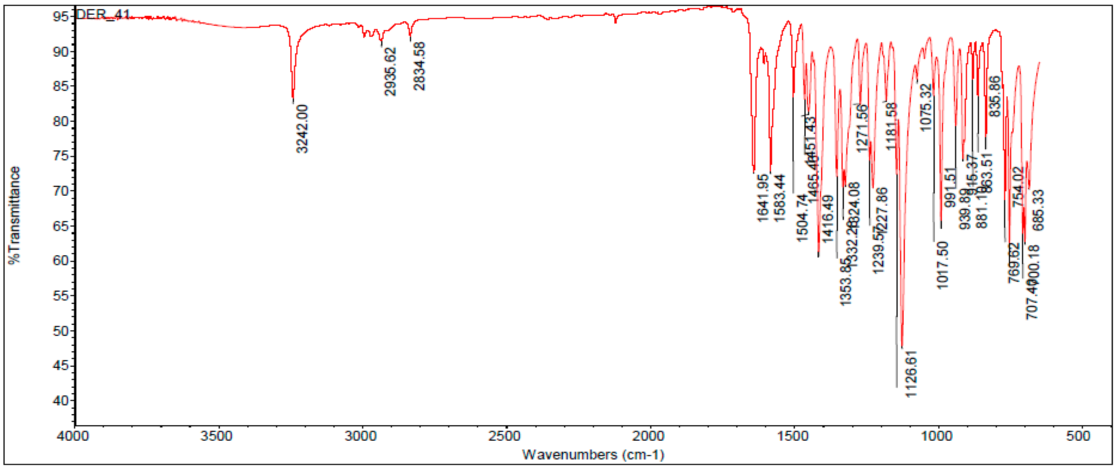

Figure S27. Absorption spectra in the infrared region of *(E)-N'*-benzylidene-3,4,5-trimethoxy-*N*-(prop-2-yn-1-yl)benzohydrazide (**9**) (ATR).

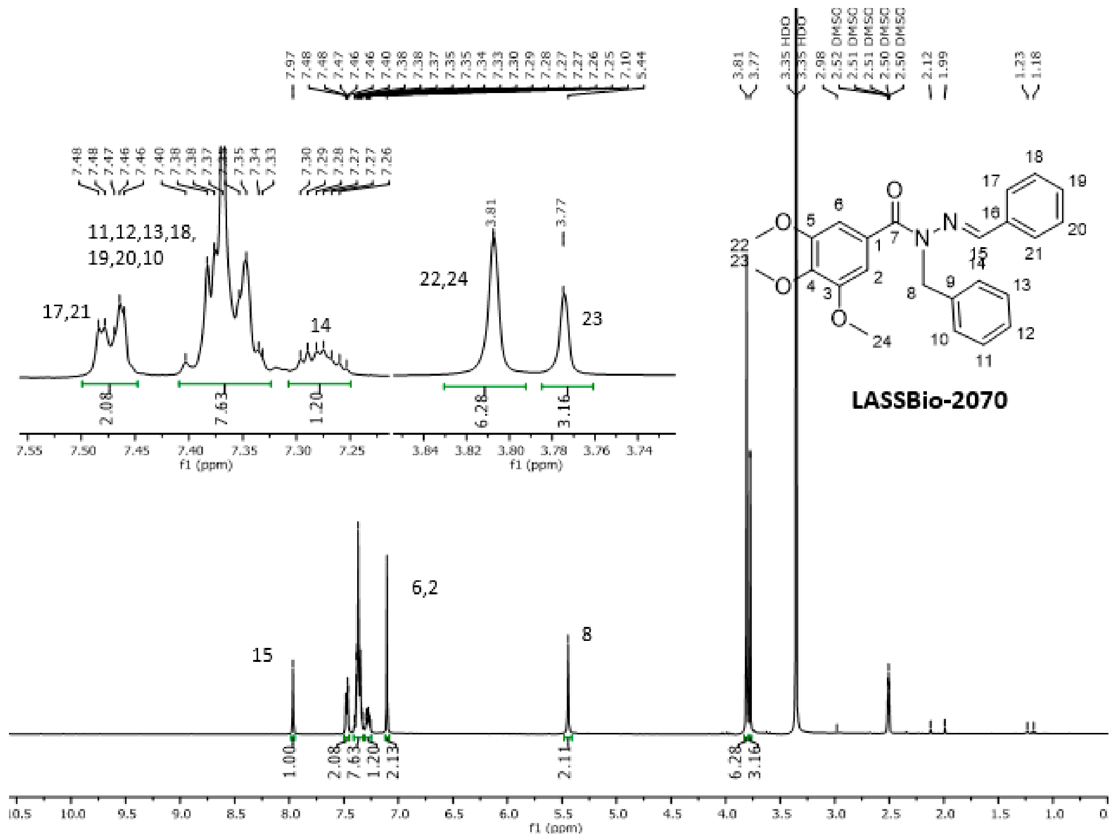

Figure S28. ^{1}H NMR spectra of *(E)-N*-benzyl-*N'*-benzylidene-3,4,5-trimethoxybenzohydrazide (**10**) (400 MHz, DMSO-d_6).

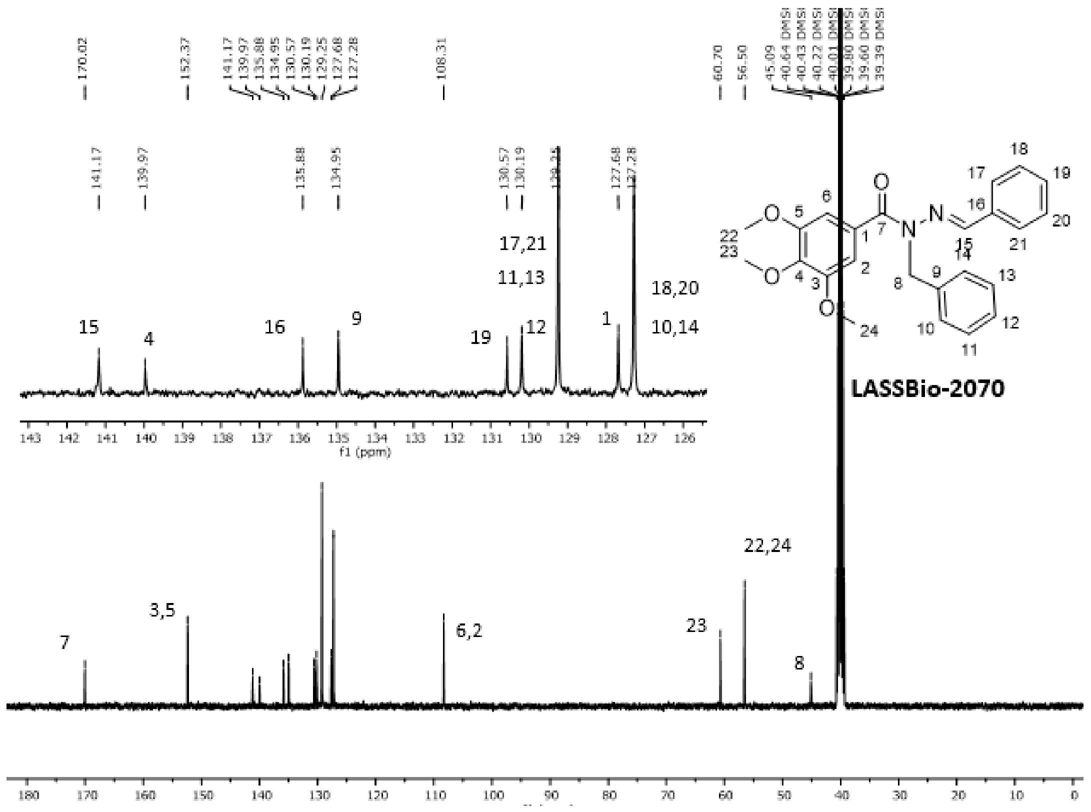

Figure S29. ^{13}C NMR spectra of *(E)-N*-benzyl-*N'*-benzylidene-3,4,5-trimethoxybenzohydrazide (**10**) (100 MHz, DMSO-*d*$_6$).

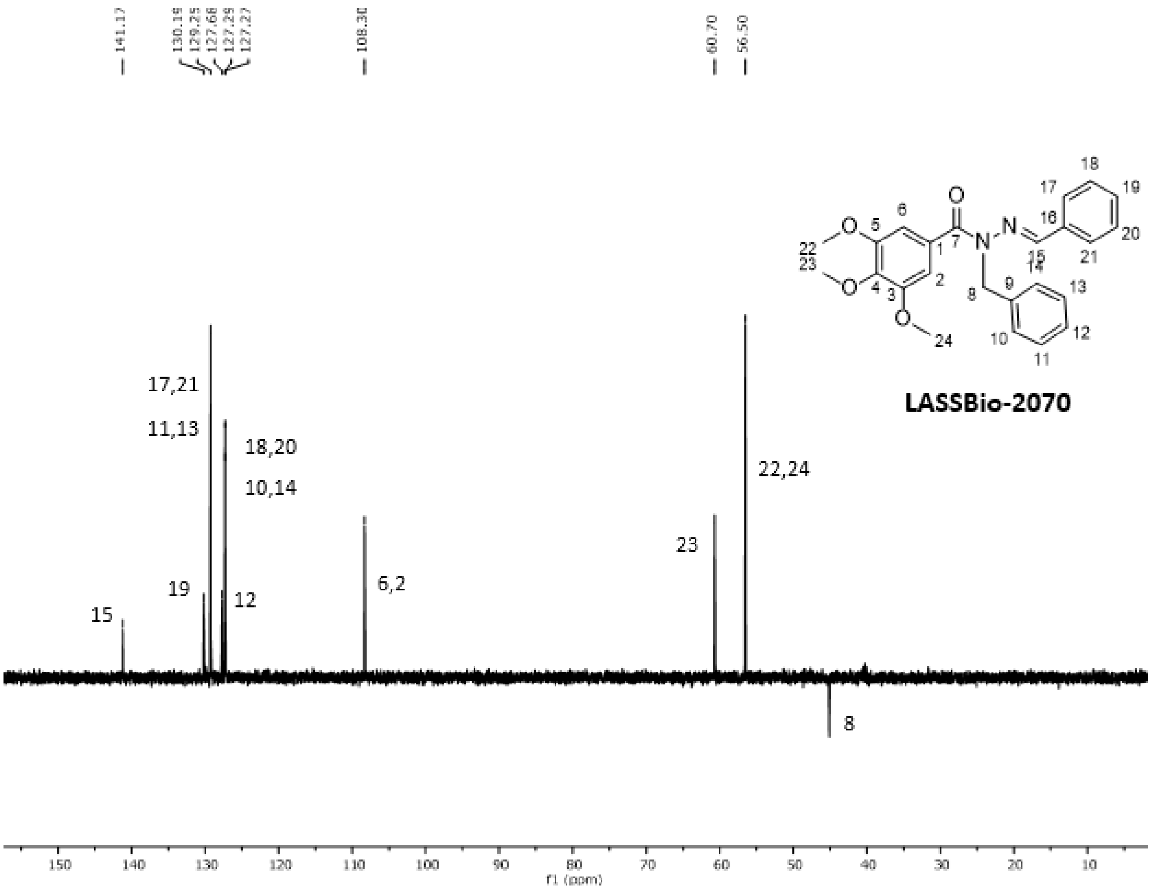

Figure S30. ^{13}C DEPT-135 NMR spectra of *(E)*-*N*-benzyl-*N*'-benzylidene-3,4,5-trimethoxybenzohydrazide (**10**) (126 MHz, DMSO-*d*$_6$).

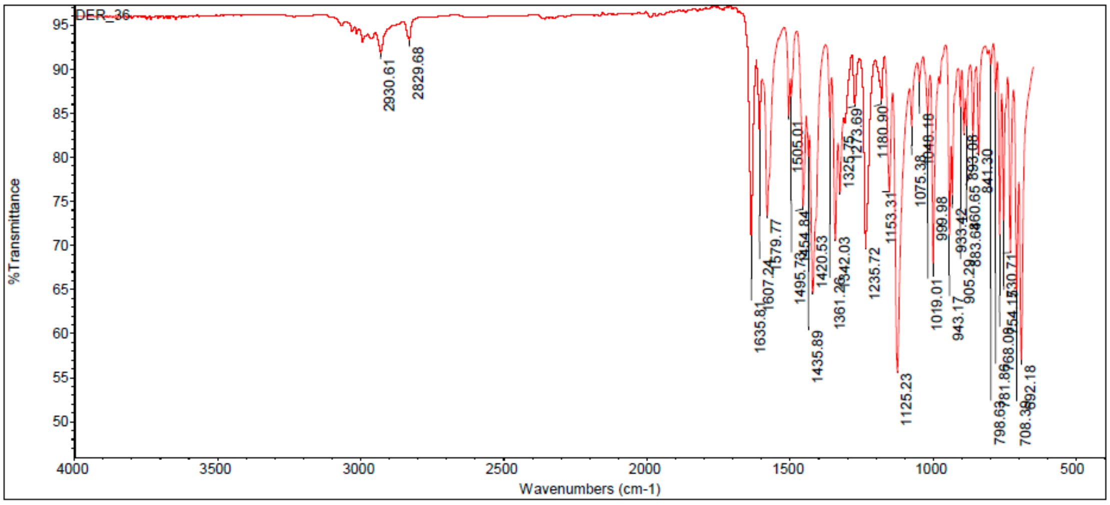

Figure S31. Absorption spectra in the infrared region of *(E)*-*N*-benzyl-*N*'-benzylidene-3,4,5-trimethoxybenzohydrazide (**10**) (ATR).

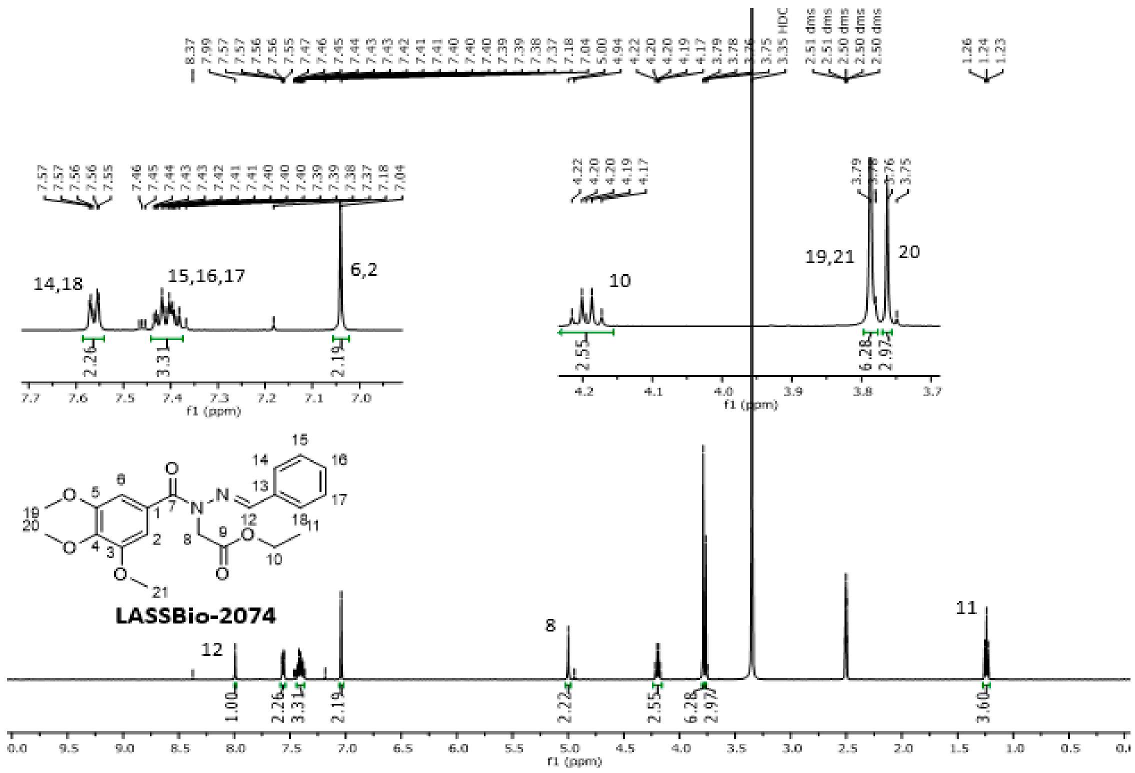

Figure S32. ^{1}H NMR spectra of Ethyl *(E)*-*N*-(benzylideneamino)-*N*-(3,4,5-trimethoxybenzoyl)glycinate (**11**) (400 MHz, DMSO-d_6).

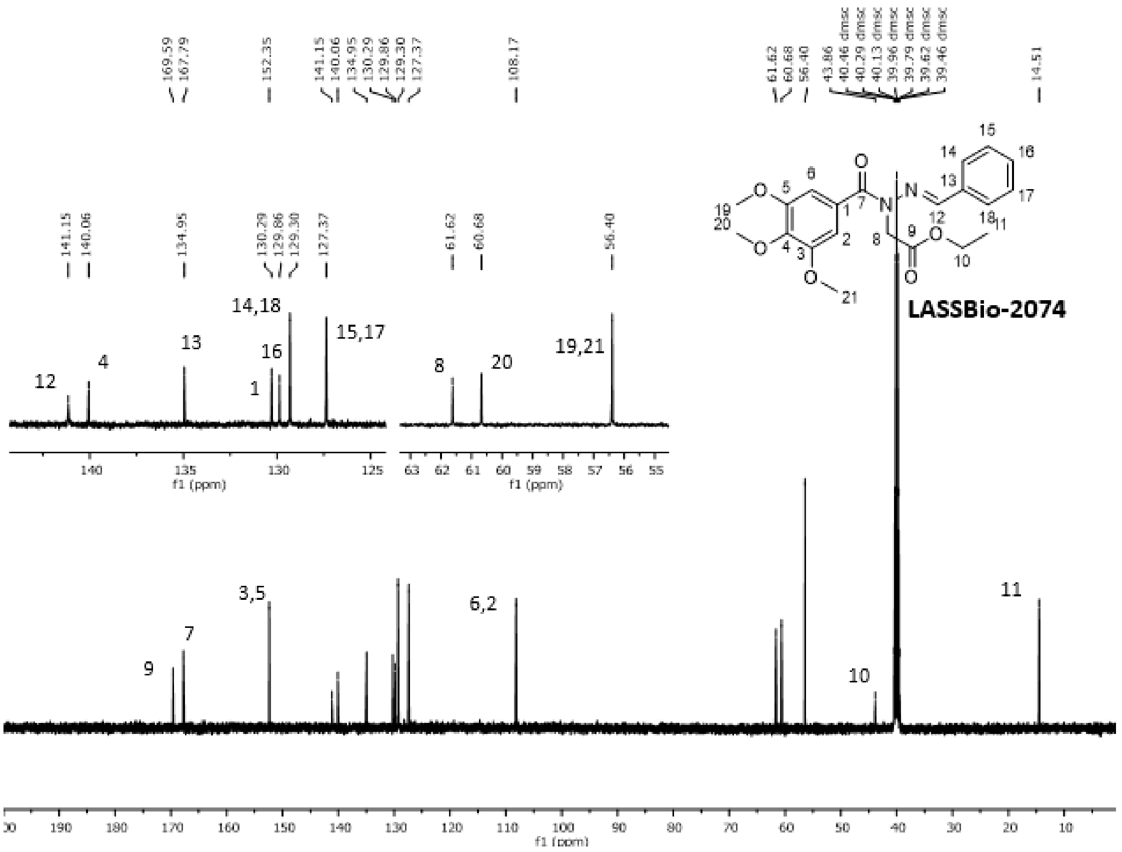

Figure S33. ^{13}C NMR spectra of Ethyl *(E)*-*N*-(benzylideneamino)-*N*-(3,4,5-trimethoxybenzoyl)glycinate (**11**) (100 MHz, DMSO-d_6).

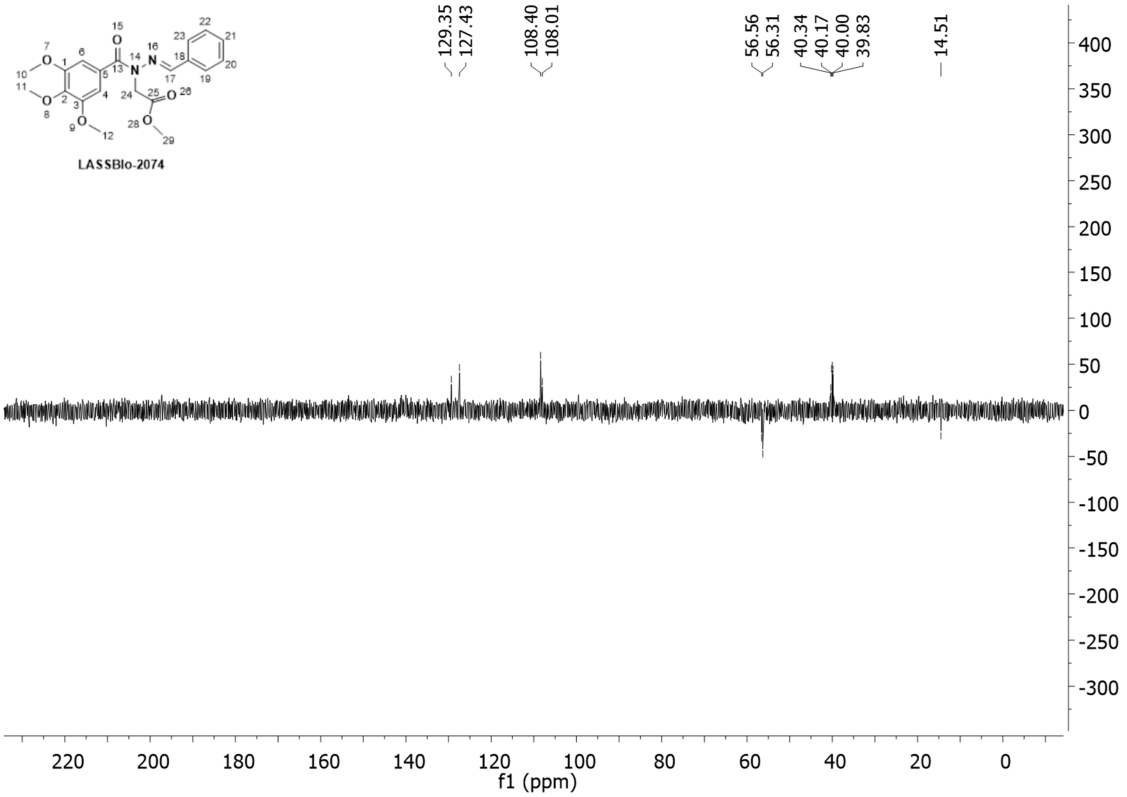

Figure S34. ^{13}C DEPT-135 NMR spectra of Ethyl *(E)-N*-(benzylideneamino)-*N*-(3,4,5-trimethoxybenzoyl)glycinate (**11**) (126 MHz, DMSO-d_6).

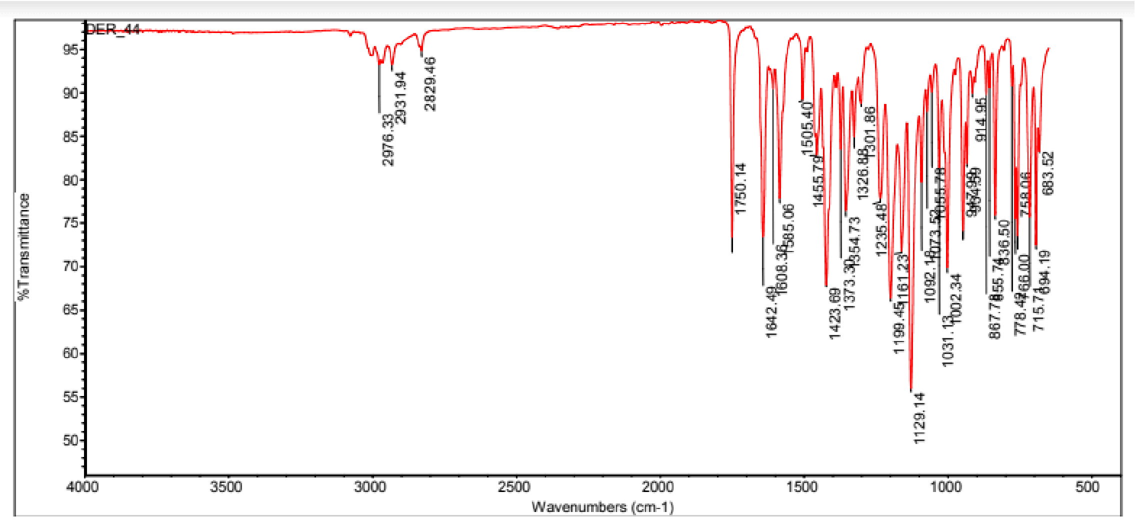

Figure S35. Absorption spectra in the infrared region of Ethyl *(E)-N*-(benzylideneamino)-*N*-(3,4,5-trimethoxybenzoyl)glycinate (**11**) (ATR).

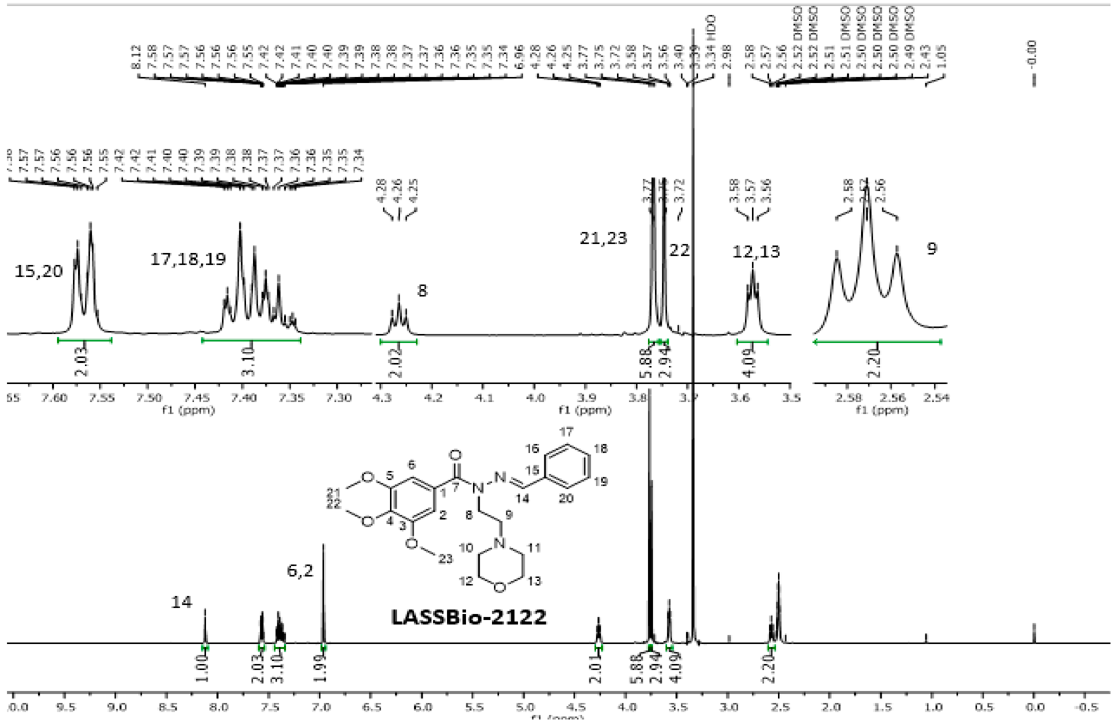

Figure S36. ^{1}H NMR spectra of *(E)-N'*-benzylidene-3,4,5-trimethoxy-*N*-(2-morpholinoethyl)benzohydrazide (**12**) (500 MHz, DMSO-*d*$_6$).

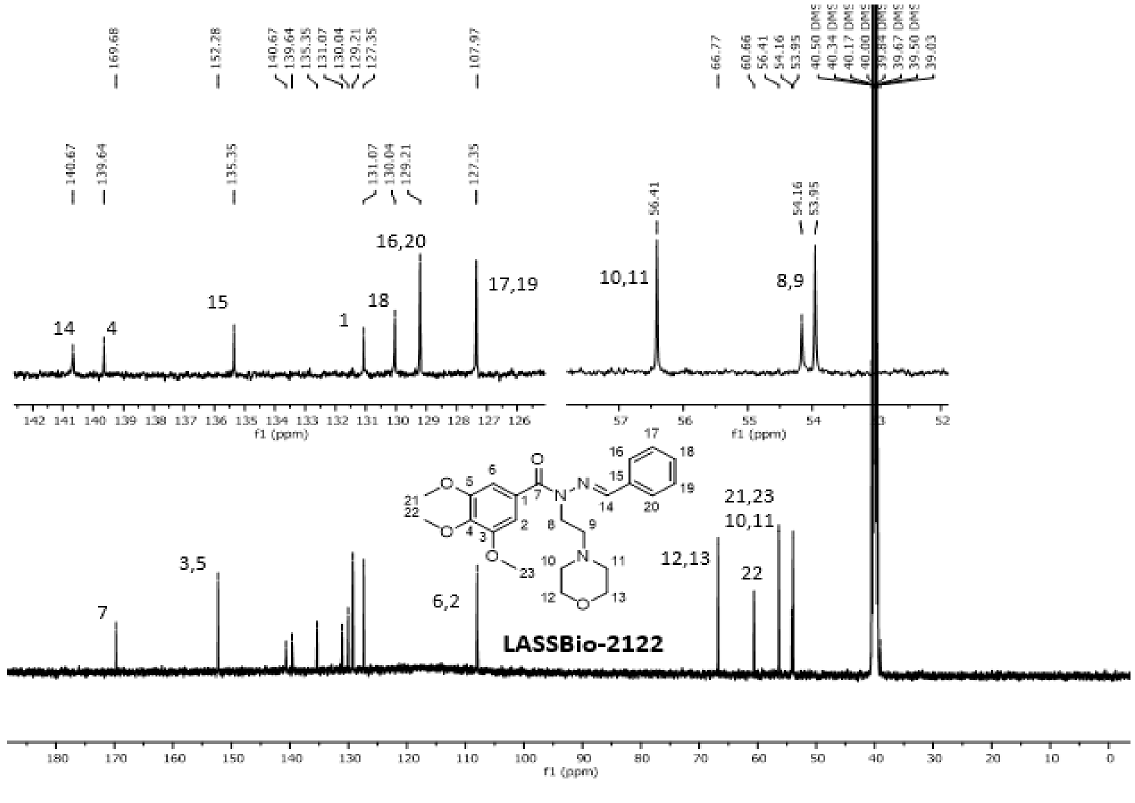

Figure S37. ^{13}C NMR spectra of *(E)-N'*-benzylidene-3,4,5-trimethoxy-*N*-(2-morpholinoethyl)benzohydrazide (**12**) (125 MHz, DMSO-*d*$_6$).

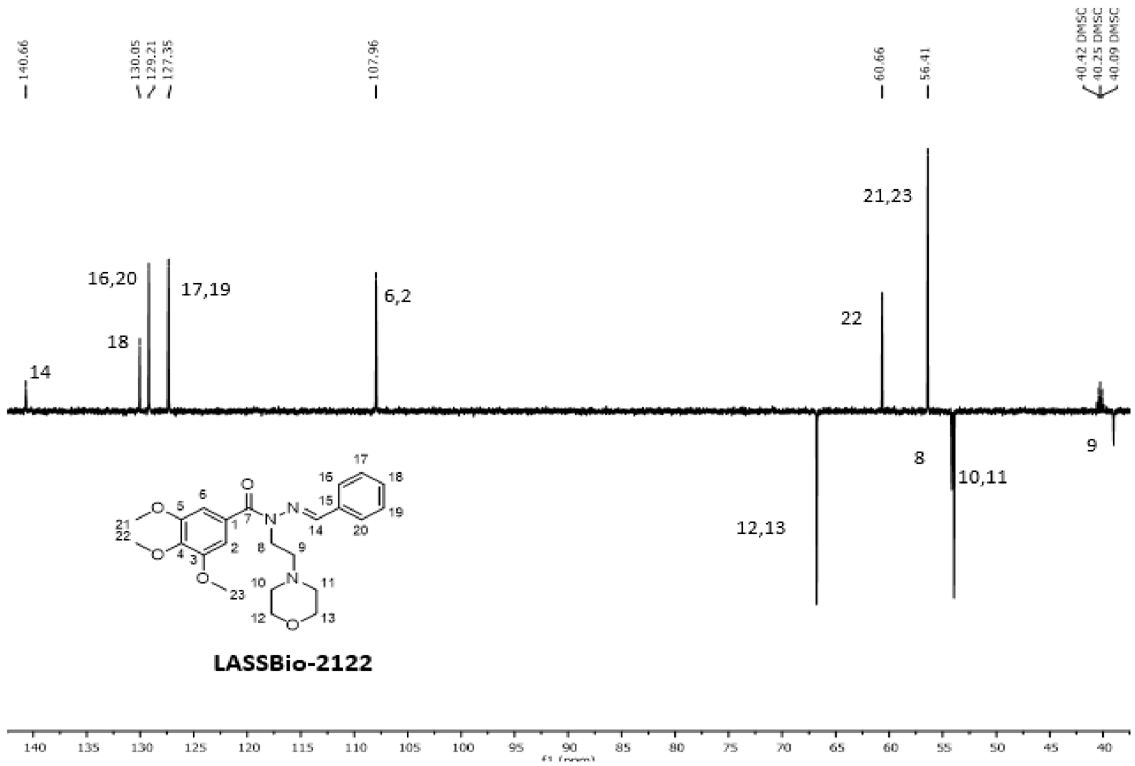

Figure S38. ^{13}C DEPT-135 NMR spectra of *(E)-N'*-benzylidene-3,4,5-trimethoxy-*N*-(2-morpholinoethyl)benzohydrazide (**12**) (126 MHz, DMSO-d_6).

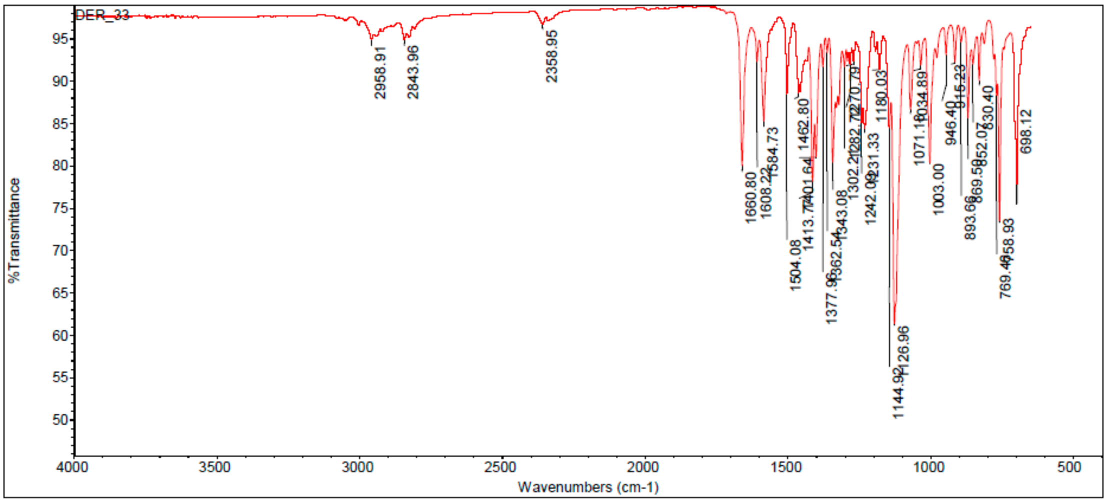

Figure S39. Absorption spectra in the infrared region of *(E)-N'*-benzylidene-3,4,5-trimethoxy-*N*-(2-morpholinoethyl)benzohydrazide (**12**) (ATR).

Compound selectivity index (SI) data at 48h by MTT

Table S1. The selectivity index (SI) of CA-4, pelitinib and *N*-acylhydrazones (**2-12**) in 48 hours

**Compounds**	**SI**				
	**Lymphocyte/ HL-60**	**Lymphocyte/ H1975**	**Lymphocyte/ H292**	**Lymphocyte/ LoVo**	**Lymphocyte/ PC-9**
**CA-4 (1)**	0.01	0.3	0.2	0.1	0.6
**Pelitinib**	0.3	0.3	2.0	0.5	2.8
**LASSBio-1586 (2)**	0.3	0.45	0.4	0.5	1.0
**LASSBio-1735 (3)**	0.09	**7.95**	**5.0**	1.6	0.6
**LASSBio-2071 (4)**	0.01	0.01	0.2	0.02	0.06
**LASSBio-2118 (5)**	1.4	2.2	**11.0**	2.7	7.6
**LASSBio-2121 (6)**	0.42	0.64	5.0	0.54	3.35
**LASSBio-2069 (8)**	2.3	0.72	**7.7**	1.5	4.3
**LASSBio-2072 (9)**	0.02	0.02	0.2	0.03	0.07
**LASSBio-2070 (10)**	0.62	3.5	**26.6**	1.2	**16.0**
**LASSBio-2074 (11)**	0.44	0.52	**4.25**	1.0	1.8
**LASSBio-2122 (12)**	0.18	0.14	2.3	0.9	0.6

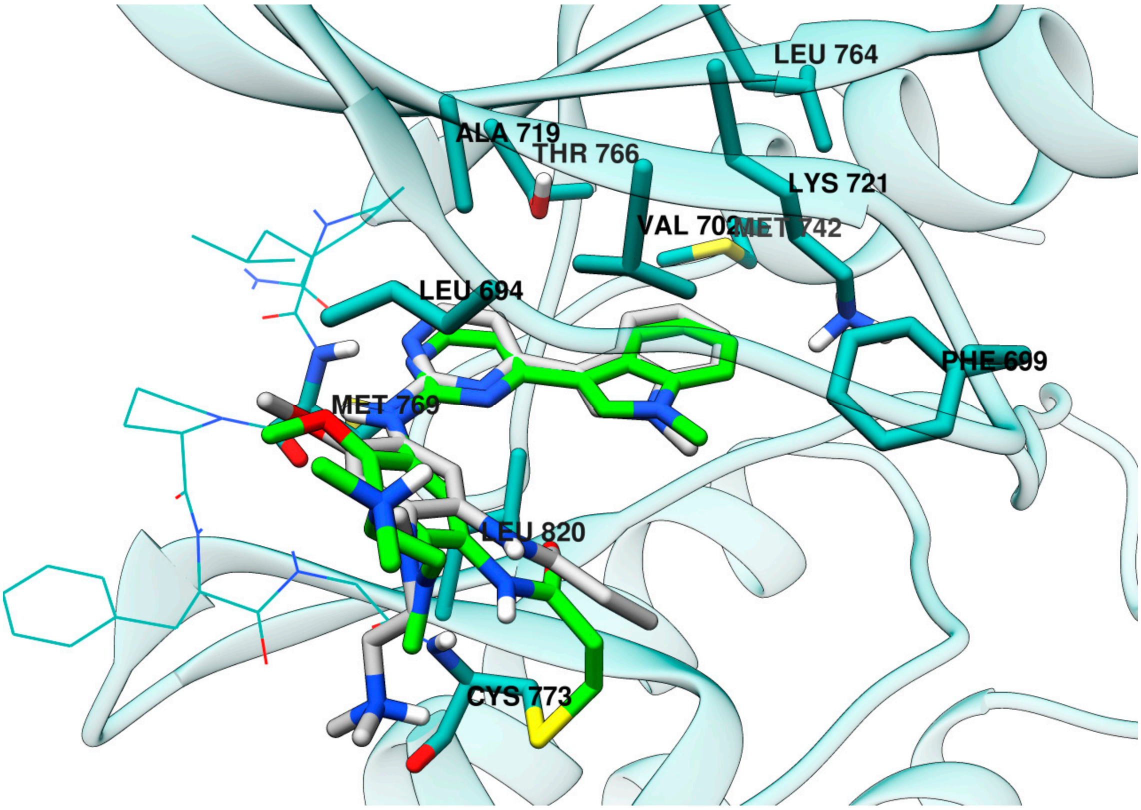

Figure S4. Interaction mode of osimertinib (covalent adduct in green, non-covalent in grey). Protein carbon atoms are represented in light blue, oxygen in red, nitrogen in blue and the distances between hydrogen bond are represented by the green line. Hydrophobic interactions are represented by yellow lines. Figures were produced with UCSF Chimera.

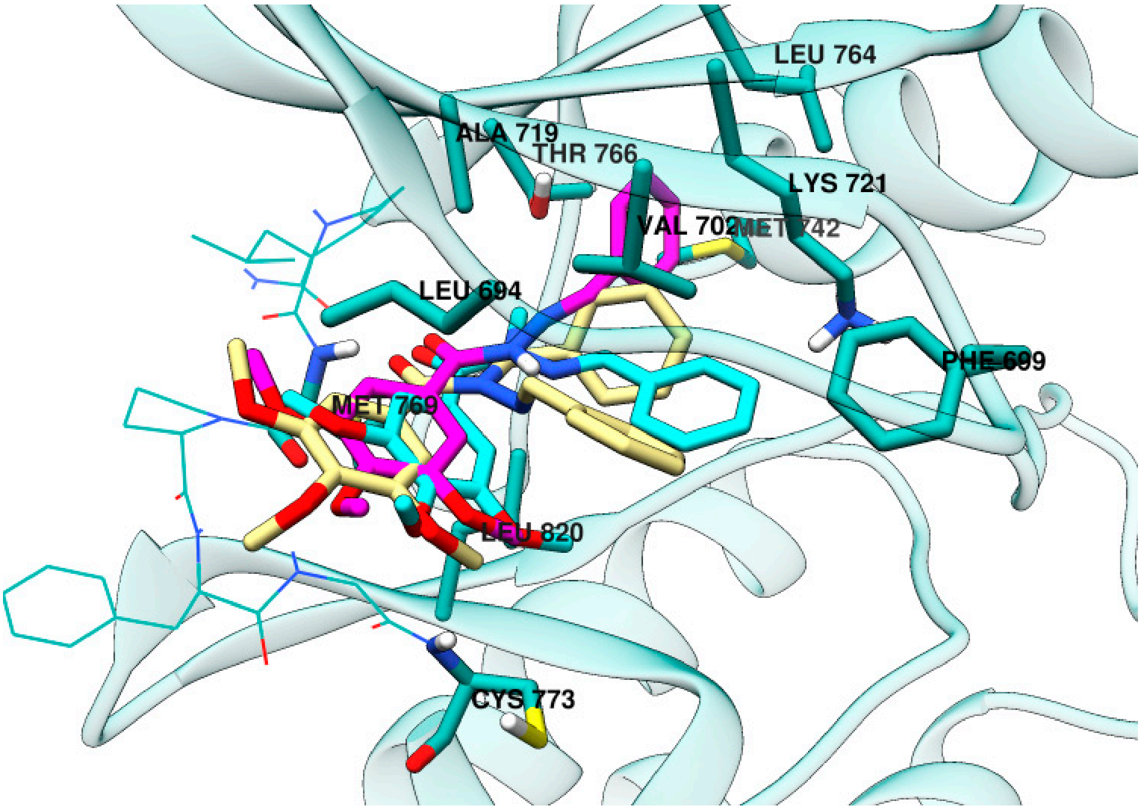

Figure S41. Superposition of LASSBio-1586 (**2**) in magenta, LASSBio-1735 (**3**) in cyan, and LASSBio-2070 (**10**) in yellow. Protein carbon atoms are represented in light blue, oxygen in red, nitrogen in blue and the distances between hydrogen bond are represented by the green line. Hydrophobic interactions are represented by yellow lines. Figures were produced with UCSF Chimera.
